# A H3K27M-targeted vaccine in adults with diffuse midline glioma

**DOI:** 10.1038/s41591-023-02555-6

**Published:** 2023-09-21

**Authors:** Niklas Grassl, Isabel Poschke, Katharina Lindner, Lukas Bunse, Iris Mildenberger, Tamara Boschert, Kristine Jähne, Edward W. Green, Ingrid Hülsmeyer, Simone Jünger, Tobias Kessler, Abigail K. Suwala, Philipp Eisele, Michael O. Breckwoldt, Peter Vajkoczy, Oliver M. Grauer, Ulrich Herrlinger, Joerg-Christian Tonn, Monika Denk, Felix Sahm, Martin Bendszus, Andreas von Deimling, Frank Winkler, Wolfgang Wick, Michael Platten, Katharina Sahm

**Affiliations:** 1 DKTK CCU Neuroimmunology and Brain Tumor Immunology, German Cancer Research Center (DKFZ), Heidelberg, Germany; 2 Department of Neurology, Medical Faculty Mannheim, MCTN, Heidelberg University, Mannheim, Germany; 3 DKFZ-Hector Cancer Institute at University Medical Center Mannheim, Mannheim, Germany; 4 Immune Monitoring Unit, German Cancer Research Center (DKFZ) and National Center for Tumor Diseases (NCT), Heidelberg, Germany; 5 Helmholtz Institute for Translational Oncology (HI-TRON) Mainz, German Cancer Research Center, Mainz, Germany; 6 Department of Neurology, University Hospital Heidelberg, Heidelberg, Germany; 7 National Center for Tumor Diseases (NCT), University Hospital Heidelberg, Heidelberg, Germany; 8 Department of Neuropathology, University Hospital Heidelberg, Heidelberg, Germany; 9 DKTK Clinical Cooperation Unit Neuropathology, German Cancer Research Center (DKFZ), Heidelberg, Germany; 10 Department of Neuroradiology, Heidelberg University Hospital, Heidelberg, Germany; 11 Department of Neurosurgery, Charité-Universitätsmedizin Berlin, Berlin, Germany; 12 Department of Neurology with Institute of Translational Neurology, University of Münster, Münster, Germany; 13 Division of Clinical Neurooncology, Department of Neurology, University Hospital Bonn, University of Bonn, Bonn, Germany; 14 Department of Neurosurgery, University of Munich LMU, Munich, Germany; 15 Institute of Cell Biology, Department of Immunology, University of Tübingen, Tübingen, Germany

**Keywords:** CNS cancer, CNS cancer, Immunization, Peptide vaccines, Cancer in the nervous system

## Abstract

Substitution of lysine 27 to methionine in histone H3 (H3K27M) defines an aggressive subtype of diffuse glioma. Previous studies have shown that a H3K27M-specific long peptide vaccine (H3K27M-vac) induces mutation-specific immune responses that control H3K27M^+^ tumors in major histocompatibility complex-humanized mice. Here we describe a first-in-human treatment with H3K27M-vac of eight adult patients with progressive H3K27M^+^ diffuse midline glioma on a compassionate use basis. Five patients received H3K27M-vac combined with anti-PD-1 treatment based on physician’s discretion. Repeat vaccinations with H3K27M-vac were safe and induced CD4^+^ T cell-dominated, mutation-specific immune responses in five of eight patients across multiple human leukocyte antigen types. Median progression-free survival after vaccination was 6.2 months and median overall survival was 12.8 months. One patient with a strong mutation-specific T cell response after H3K27M-vac showed pseudoprogression followed by sustained complete remission for >31 months. Our data demonstrate safety and immunogenicity of H3K27M-vac in patients with progressive H3K27M^+^ diffuse midline glioma.

## Abstract (web summary)

In this compassionate use study, treatment of adult patients with H3K27M-mutant diffuse midline glioma with a long peptide vaccine targeting H3K27M led to vaccine-induced peripheral T cell immune responses and encouraging clinical efficacy in the majority of patients, including a durable complete response.

## Main

H3K27M^+^ diffuse midline gliomas (DMGs) are aggressive, incurable primary central nervous system (CNS) tumors in children and young adults^1^. They are characterized by a clonal and mutually exclusive substitution of lysine 27 to methionine (K27M) in canonical (*H3.1*/*H3.2*) or noncanonical (*H3.3*) histone H3 (ref. ^2^) in anatomically distinct oligodendrocyte precursor cells^3,4^. As these tumors mainly form in midline CNS structures, surgical treatment options remain limited^5,6^. Response to chemoradiation is poor and palliative radiotherapy remains the only standard-of-care treatment with proven benefit^6^, resulting in a median overall survival (OS) between 10 and 15 months after initial diagnosis^7^. Immune checkpoint inhibitors (ICIs), such as PD-1 blockade are successfully used in combinatorial immunotherapeutic approaches in high-grade gliomas^8^; however, in DMG intratumoral heterogeneity^9^, low PD-L1 expression^10^, low mutational burden^11^ and the nature of chemotherapy-induced mutations^12,13^ may explain why no survival benefit has been observed using ICI monotherapy^14^ so far, though several clinical trials investigating the efficacy and safety of PD-1 blockade are ongoing (NCT02359565, NCT02793466, NCT03130959 and NCT01952769).

New immunotherapeutic approaches with specificity for DMG include disialoganglioside GD2-targeting chimeric antigen receptor (CAR) T cell therapy^15^, the oncolytic virus DNX-2401 (ref. ^16^) and peptide vaccination^17–19^. A short H3.3K27M_26–35_ peptide vaccine induced H3.3K27M-reactive CD8^+^ T cells in human leukocyte antigen (HLA)-A*02:01^+^ patients with newly diagnosed H3.3K27M^+^ DMG^17^. Whether such HLA-A*02:01-restricted CD8^+^ T cells recognize and kill HLA-A*02:01^+^ tumor cells expressing and processing endogenous H3.3K27M remains controversial^17,19^. We have previously shown that a long H3K27M_14–40_ peptide vaccine, H3K27M-vac, induced CD4^+^ T cell-mediated immune responses in a major histocompatibility complex (MHC)-humanized mouse tumor model^18^. Here, we present a first-in-human administration of H3K27M-vac to eight patients with progressive H3K27M^+^ DMG.

## Results

**Fig. 1:**
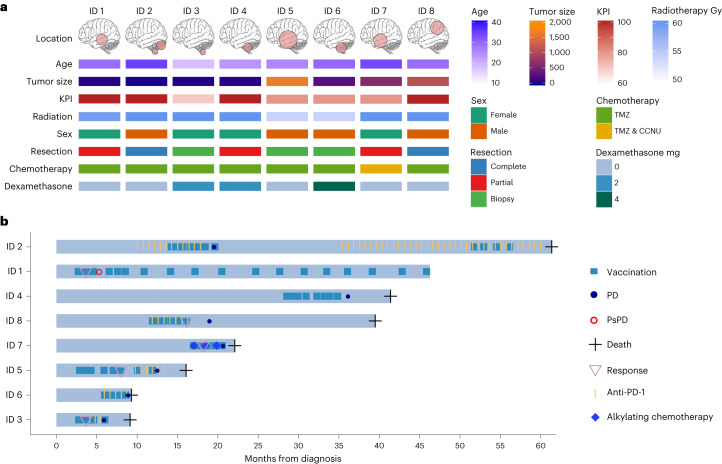
Patient characteristics at baseline and individual disease trajectories. **a**, Baseline characteristics of eight patients with progressive DMG H3K27M^+^ before initiation of treatment with H3K27M-vac. Age specified in years, tumor size measured as product of maximal orthogonal diameter on contrast-enhanced T1-weighted MRI sequences (mm^2^); cumulative dose of intensity-modulated radiotherapy measured in Gy; TMZ, temozolomide (75 mg m^−2^ body surface area (BSA)) daily during radiotherapy; CCNU, lomustine (110 mg m^−2^ BSA d1, TMZ mg m^−2^ BSA d2–6, q42d for six cycles); oral dexamethasone intake in mg d^−1^. Brain illustration taken from Adobe Stock Standard under License ID 222738500. **b**, Swimmer plot depicting clinical course since initial diagnosis, vaccine administration and time point of first H3K27M-specific immune responses in peripheral blood (*n* = 8 patients). Source data

A total of eight adult patients with progressive, histologically confirmed H3K27M^+^ DMG after standard therapy options and not eligible to be enrolled in the currently ongoing multicenter, phase I clinical trial (NCT04808245) received H3K27M-vac on a compassionate use basis. Four patients were female and four patients were male (Fig. 1a), mean patient age was 28.0 ± 5.3 years (mean ± s.d.) and Karnofsky performance index (KPI) was at least 70% for all patients. All patients had unequivocal progressive disease (PD) as assessed by response assessment in neuro-oncology (RANO) criteria before the start of vaccinations. Tumors were located in the thalamus (*n* = 3), the pons (*n* = 2), the spinal cord (*n* = 2) and the parietal lobe (*n* = 1), with one patient having multilocular disease in the cerebellum and the lumbar spinal cord. Two patients had undergone complete resection, three patients had partial resection and three patients had biopsies upon initial radiographic diagnosis. At first dosing, two patients took dexamethasone at a dose of 2 mg d^−1^ and one patient took 4 mg d^−1^ (Extended Data Table 1). All eight patients had previously received radiotherapy in 30 fractions to a total dose ranging from 54 to 60 Gy as well as chemotherapy with temozolomide. One patient (ID 7) received lomustine q42d following first PD and continued this therapy concomitant to vaccinations. Median tumor size as judged by the product of maximal orthogonal tumor diameter at baseline was 407.8 ± 589.4 mm^2^ (median ± s.d.).

### H3K27M-vac was well tolerated

**Fig. 2:**
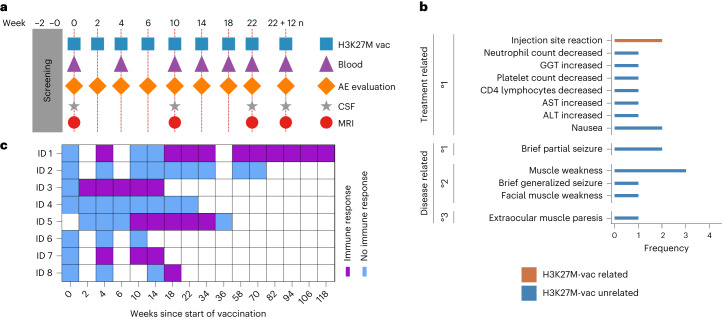
Treatment schedule, safety and immunogenicity of H3K27M-vac. **a**, Treatment scheme for H3K27M-vac administration. **b**, Treatment-related AEs occurring in the observation period graded by CTCAE v.5.0. Two injection site reactions were related to H3K27M-vac and the remaining AEs were either judged to be related to concomitant medication or disease. GGT, gamma-glutamyltransferase; ALT, alanine aminotransferase; AST, aspartate aminotransferase. **c**, T cell immune responses as a function of time measured by difference in mean spot-forming units (s.f.u.) in IFN-γ ELISpot assay between 4 × 10^5^ peripheral blood mononuclear cells (PBMCs) stimulated with H3-mut and H3-wt control peptide. Source data

Patients received subcutaneous injections of H3K27M-vac bi-weekly for 6 weeks followed by monthly administration for 4 months and quarterly thereafter until PD (Fig. 1b, Fig. 2a and Supplementary Fig. 1). Five patients (62.5%) received H3K27M-vac in combination with anti-PD-1 dependent on the treating physician’s discretion. Before each vaccination, adverse events (AEs) were assessed according to the Common Terminology Criteria for Adverse Events (CTCAE) v.5.0. In addition, the treatment schedule included monthly blood sampling for immune monitoring for 6 months and every 3 months thereafter as well as radiographic assessment every 3 months. Analysis of cerebrospinal fluid (CSF) was performed if clinically indicated (Fig. 2a). The duration of H3K27M-vac treatment ranged from 78 to 1,295 d (median 158 d) and the duration of observation since the start of H3K27M-vac administration ranged from 191 to 1,414 d (median 391 d). Patients received a median of 8 ± 4.9 (median ± s.d.) vaccinations. One patient (ID 2) discontinued treatment with H3K27M-vac after eight vaccinations, but resumed treatment 20 months later. No regimen-limiting toxicity was observed during the observation period. Two patients (25%) experienced CTCAE grade 1 injection site reactions that were attributed to the treatment with H3K27M-vac (Fig. 2b). Eight other CTCAE grade 1 events in the observation period were judged to be treatment related, but unrelated to H3K27M-vac; higher grade treatment-related toxicities have not occurred.

### H3K27M-vac induced neoepitope-specific immune responses

**Extended Data Fig. 1:**
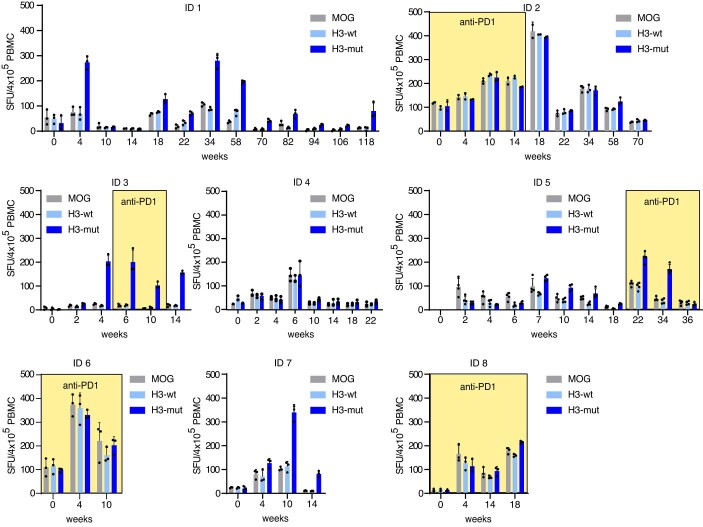
IFNγ-ELISpot responses for all 8 patients over time. Spot forming units (SFU) per 4 × 10^5^ PBMCs after restimulation with MOG, H3-wt and H3-mut. Dots mark individual data points, bar plots the mean and error bars the SD (n = 3 BIE for all patients, conditions and time points except for ID2 week 0, 4, 14, 18, where n = 2 BIE). Yellow background indicates the time period of concomitant anti-PD-1 therapy. Source data

**Extended Data Fig. 2:**
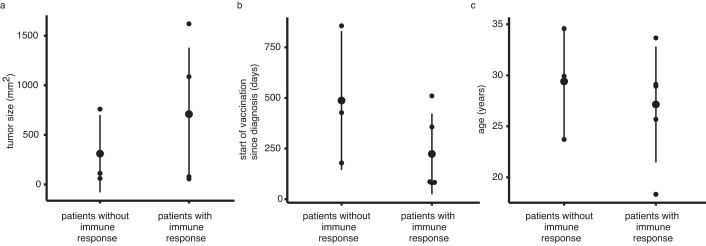
Distribution of tumor size, beginning of vaccination and age between patients with immune response and patients without response at baseline. Distribution of tumor size in mm^2^ determined by product of maximal orthogonal diameters on T1 weighted contrast enhanced MRI imaging (**a**) time between histological diagnosis and start of vaccination in days (**b**) and patient age in years at start of vaccination (**c**) between patients with H3K27M specific immune response and patients without H3K27M specific immune response. Source data

We observed H3K27M-vac-induced peripheral T cell immune responses defined by mutation-specific interferon (IFN)-γ enzyme-linked immunosorbent spot (ELISpot) responses detected in peripheral blood in five of eight treated patients (62.5%; Fig. 2c). The median time to first detectable H3K27M-specific immune response was two vaccinations (median, interquartile range (IQR) 2–4), corresponding to 4 weeks (median, IQR 4–10) since the start of treatment. In four out of five responding patients, the specific ELISpot responses detected in peripheral blood decreased over time (Extended Data Fig. 1). In this eight-patient cohort, there was no apparent association between H3K27M-specific peripheral immune response and age (*P* = 0.60), sex (*P* = 0.46), KPI (*P* = 0.75), extent of resection (*P* = 0.94), tumor size (*P* = 0.08), time from histological diagnosis to start of vaccination (*P* = 0.06), concomitant anti-PD-1 treatment (*P* = 0.57), dexamethasone intake at baseline (*P* = 0.15) or HLA allelotype (Extended Data Table 2 and Extended Data Fig. 2a–c).

### Patients with immune responses showed radiographic improvement

**Fig. 3:**
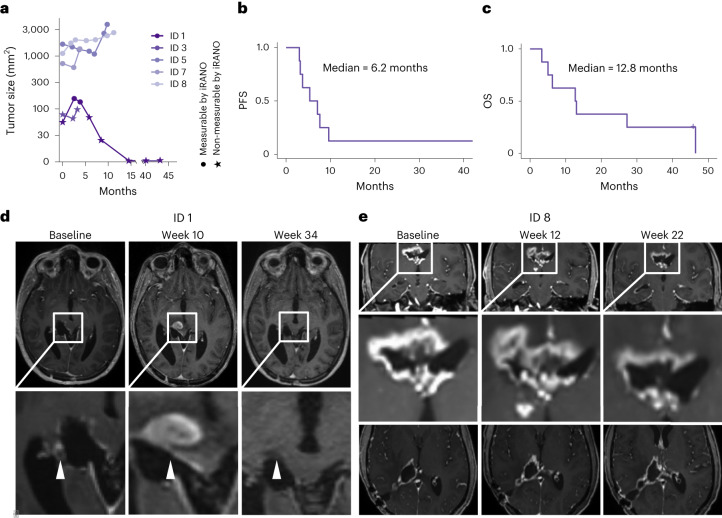
Clinical response to H3K27M-vac. **a**, Tumor size in mm^2^ as a function of time in months from start of vaccination. Size determined by product of maximal orthogonal diameters on T1-weighted contrast-enhanced MRI imaging. Dots indicate measurements that are considered measurable by iRANO criteria (cerebral lesion with diameter >10 mm). **b**,**c**, PFS (**b**) and OS (**c**) since the start of vaccination. **d**, T1-weighted with contrast enhancement (CE) MRI sequences of PsPD of patient ID 1 at baseline, week 10 and week 34. White arrows indicate tumor lesion with PsPD at week 10. **e**, T1-weighted with CE MRI series of patient ID 8 with early progression between baseline and week 12 followed by disease stabilization concurrent to first detectable H3K27M-specific immune response in peripheral blood in week 18. Source data

**Extended Data Fig. 3:**
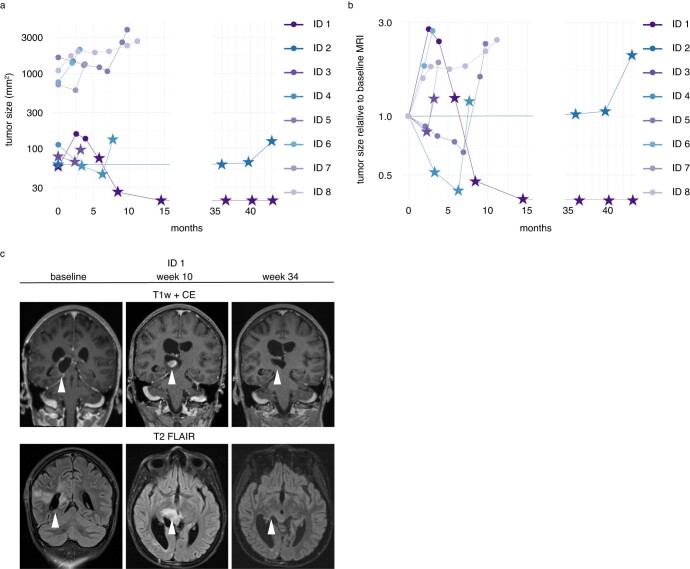
Longitudinal course of all tumor diameters and pseudoprogression in ID 1. **a, b** Tumor size in mm^2^ (**a**) and relative to baseline before start of vaccination (**b**) as a function of time in months from start of vaccination. Size determined by product of maximal orthogonal diameters on T1 weighted contrast enhanced (CE) MRI imaging. Dots indicate measurements that are considered measurable by iRANO criteria (that is cerebral lesion with diameters > 10 mm) and stars non-measurable lesions by iRANO criteria. **c**, Coronary T1-weighted CE sequences (top) and fluid-attenuated inversion recovery sequences (bottom) of PsPD of patient ID 1 at baseline, week 10 and week 34. White arrows indicate tumor lesion with PsPD in week 10. Source data

Transient radiographic improvement defined as reduction of the axial contrast-enhancing tumor area was observed in six patients and occurred shortly after first detection of H3K27M-specific immune responses in all five patients with immune response (Fig. 3a, Extended Data Fig. 3a,b and Supplementary Figs. 2–9). Median progression-free survival (PFS) after start of vaccination across all eight patients was 6.2 months and median OS was 12.8 months (Fig. 3b,c). One patient treated with H3K27M-vac without concomitant anti-PD-1 therapy (ID 1) showed radiographic pseudoprogression (PsPD) according to immunotherapy response assessment in neuro-oncology (iRANO) criteria within 6 weeks after first detection of mutation-specific peripheral immune response (Fig. 3a,d, Extended Data Fig. 3c and Supplementary Fig. 2). Another patient with large contrast-enhancing tumor mass at baseline and concomitant anti-PD-1 therapy (ID 8) showed an early radiographic progression followed by disease stabilization from week 22 onwards in line with a latency of 18 weeks until first detection of a mutation-specific peripheral immune response (Fig. 3e and Supplementary Fig. 9).

### H3K27M neoepitope colocalized with HLA class II-DR

**Fig. 4:**
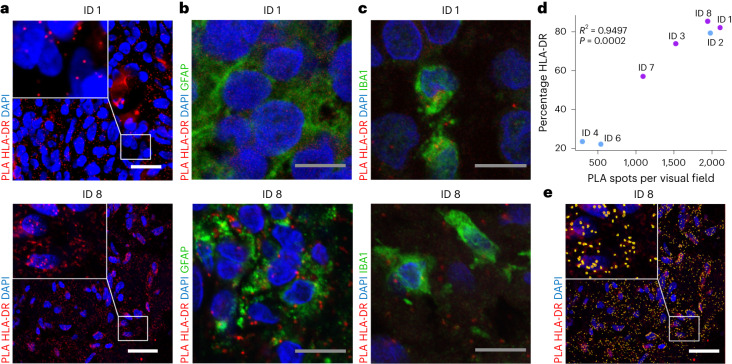
H3K27M neoepitope colocalizes with HLA class II-DR on tumor cells and myeloid cells. **a**–**c**, PLA of primary tumor tissue of patient ID 1 (top) and ID 8 (bottom) with H3K27M and HLA-DR antibodies (red) in combination with 4′,6-diamidino-2-phenylindol (DAPI) nuclear staining (blue) alone (**a**), co-staining with GFAP (green) (**b**) and co-staining with IBA1 (green) (**c**). All PLAs were repeated independently twice with similar results. Scale bar in white, 30 μm; in gray, 10 μm. **d**, Pearson correlation of PLA spots per visual field with immunohistochemistry score of HLA-DR expression across seven patients with available FFPE tissue. A two-sided *t*-test was used. **e**, Result of automated segmentation following rolling ball background subtraction, filtering with Gaussian blur and maxima detection. Scale bar in white, 30 μm. Source data

**Extended Data Fig. 4:**
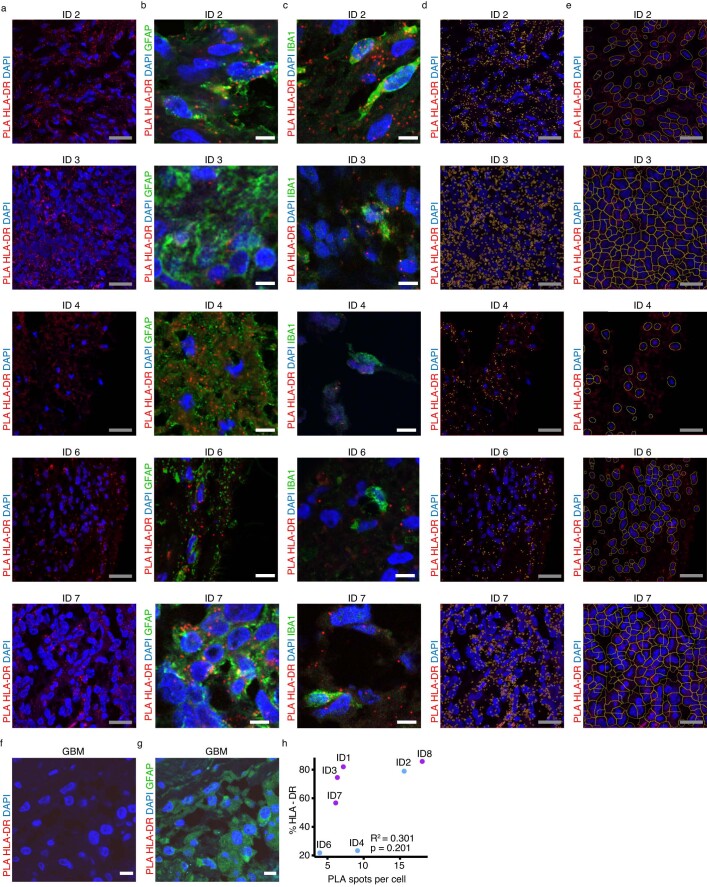
H3K27M neoepitope co-localization with HLA class II-DR on tumor cells and myeloid cells in patients ID 2, ID 3, ID 4, ID 6 and ID 7. **a–c**, Proximity ligation assay (PLA) of primary tumor tissue of patient ID 2, ID 3, ID 4, ID6 and ID7 from top to bottom with H3K27M and HLA-DR antibodies (red) in combination with 4’,6-Diamidino-2-phenylindol (DAPI) nuclear staining (blue) alone (a), co-staining with glial fibrillary acidic protein (GFAP) (green) (b) and co-staining with ionized calcium-binding adapter molecule 1 (IBA1) (green) (c). Scale bar in white = 30 μm; in gray = 10 μm. **e, f**, Automated segmentation of PLA spots (e) and nuclei (f) following rolling ball background subtraction, filtering with gaussian blur and maxima detection of visual field in a. Scale bar in gray = 10 μm. **f, g**, PLA as in a and co-staining as in b of H3-wildtype glioblastoma. Scale bar in white = 30 μm. **h**, Pearson correlation of PLA spots per cell with IHC score of HLA-DR expression across 7 patients with available FFPE tissue. Two-sided t-Test was used. Source data

**Extended Data Fig. 5:**
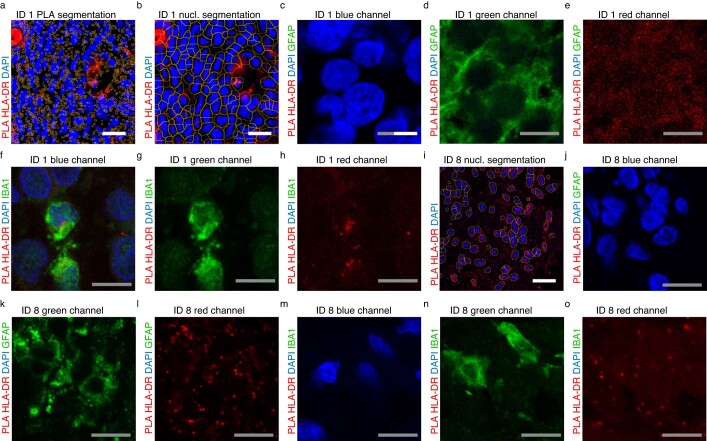
PLA segmentation and single channels of PLA images of patient ID 1 and ID 8. **a,b,i**, Automated segmentation of PLA spots (a) and nuclei (b, i) of ID 1 and ID 8 following rolling ball background subtraction, filtering with gaussian blur and maxima detection. Scale bar in white = 30 μm. **c-h, j-o**, Single channels of PLA with co-stainings as indicated on the left for ID 1 and ID 8 from images in Fig. 4a–c. Scale bar in gray = 10 μm.). All PLAs were repeated independently two times with similar results. Source data

Proximity ligation assay (PLA) of formalin-fixed paraffin-embedded (FFPE) primary tumor tissue of seven patients for which tissue was available demonstrated that the H3K27M neoepitope colocalizes with HLA class II-DR expressed by glial fibrillary acidic protein (GFAP)-expressing tumor cells as well as single ionized calcium-binding adaptor molecule 1 (IBA1)-positive professional antigen-presenting cells (APCs) in all seven patients (Fig. 4a–c and Extended Data Figs. 4a–c and 5c–h,j–o), suggesting presentation of the H3K27M neoepitope and restimulation of H3K27M-specific tumor-infiltrating HLA-DR-restricted T cells. Immunohistochemistry showed a clear interindividual heterogeneity of MHC class II-DR expression ranging from 22% to 85% positive cells as well as PLA signal intensity (295–2,100 spots per visual field) with the two patients experiencing the most favorable outcome after detection of a H3K27M-specific peripheral immune response (ID 1 and ID 8) showing top scores of 82% and 85% MHC class II-DR positive cells and 2,100 and 1,937 PLA spots per visual field, respectively (Fig. 4d,e, Extended Data Figs. 4h and 5a,b,i and Supplementary Fig. 10).

### Mutation-specific immune responses were CD4^+^ T cell-dominated

**Fig. 5:**
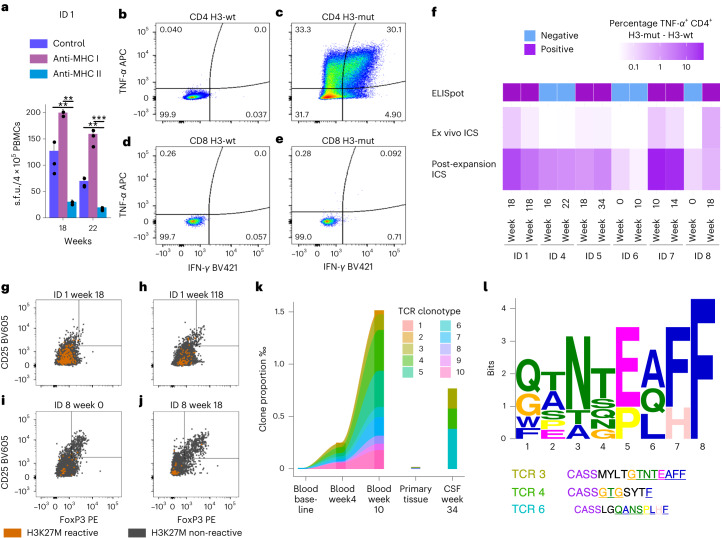
H3K27M-specific immune responses are CD4+ T cell-mediated. **a**, Suppression of H3K27M-specific IFN-γ-ELISpot response by anti-MHC class II antibody (anti-MHC II) (*n* = 2 biologically independent experiments (BIEs)), but not by anti-MHC class I antibody (anti-MHC I) (*n* = 3 BIE) compared to baseline (*n* = 3 BIE) 18 (*P* = 0.002; *P* = 0.008, top to bottom) and 22 (*P* = 0.001; *P* = 0.006, from top to bottom) weeks since start of H3K27M-vac treatment in patient ID 1. Two-sided *t*-test, not adjusted for multiple comparisons. Dots mark individual data points, bar plots show the mean and error bars indicate the s.d. ** signifies *P* < 0.01, *** signifies *P* < 0.001. **b**–**e**, Flow cytometry-based intracellular IFN-γ and tumor necrosis factor (TNF)-α detection in H3K27M-peptide expanded PBMCs restimulated with H3-wt (**b**,**d**) or H3-mut (**c**,**e**), gated on CD4^+^ (**b**,**c**) and CD8^+^ (**d**,**e**) T cell subsets. **f**, Difference in percentage of TNF-α-expressing cells among all CD4^+^ T cells between T cells stimulated with H3-mut and H3-wt either directly (ex vivo intracellular cytokine staining (ICS)) or following expansion of T cells with H3K27M peptide (post-expansion ICS). Samples were analyzed from ID 1, ID 4, ID 5, ID 6, ID 7 and ID 8 at the weeks indicated. ELISpot responses in the first column are displayed as in Fig. 1d. **g**–**j**, H3K27M-reactive, TNF-α^+^ CD4^+^ T cells (orange) among all CD4^+^ T cells (gray) did not comprise CD25^+^FoxP3^+^ regulatory T cells. Depicted are ex vivo ICS data from patient ID 1 week 18 (**g**) and week 118 (**h**) as well as patient ID 8 week 0 (**i**) and week 18 (**j**). **k**, Clonotype proportion of the ten most abundant H3K27M-vac expanded CD4^+^ T cells among all sequenced T cells in primary tissue, CSF and peripheral blood across different time points of patient ID 1. **l**, Motif plot of sequence similarities of the CDR3β region of top ten TCRs in **k** after removal of recurring CAS sequence in all ten TCRs. Overlap of CDR3β of TCR3, TCR4 and TCR6 detected in CSF with motif is indicated by color and underline. Source data

In vitro restimulation of peripheral CD4^+^ and CD8^+^ T cells with H3 mutant peptide (H3-mut) or wild-type peptide (H3-wt) revealed that H3K27M-specific immune responses are CD4^+^ T cell-mediated and can be suppressed by MHC class II-, but not MHC class I-blocking antibodies (Fig. 5a–e). Intracellular cytokine staining of peptide-stimulated PBMCs confirmed presence of H3K27M-specific CD4^+^ T cell responses with no evidence of H3K27M-vac-induced CD25^+^FoxP3^+^ regulatory T cells across multiple patients and time points (Fig. 5f–j and Supplementary Figs. 11–14). After PsPD, three out of the top ten vaccine-induced, H3K27M-expanded CD4^+^ T cell receptor (TCR) clonotypes from peripheral blood showing sequence similarities of the CDR3β region were detectable in the CSF of patient ID 1, who subsequently went into sustained complete remission for >31 months (Fig. 5k,l).

## Discussion

This first-in-human treatment with H3K27M-vac provides evidence of safety and immunogenicity against the clonal driver mutation H3K27M in patients with recurring H3K27M^+^ DMG. While this small cohort of patients with advanced-stage DMG, individual concomitant anti-PD-1 treatment and limited availability of biomaterials, including post-treatment tumor tissue limits robust conclusions on overall efficacy, the median OS of 12.8 months following a H3K27M-specific immune response and the fact that one patient exhibited sustained complete remission for >31 months are encouraging. Three general conclusions for neoepitope-targeting peptide vaccines for the treatment of diffuse gliomas can be drawn from these results.

First, the use of long peptides, such as the 27-mer H3K27M-vac, is safe irrespective of concomitant anti-PD-1 therapy and suitable to induce mutation-specific CD4^+^ T cell responses in patients with H3K27M^+^ DMG. The induction of CD4^+^ T cell-dominated immune responses by long mutation-specific peptide vaccines is similar to that observed in 28 of 30 patients in a first-in-human phase I trial of the long peptide vaccine IDH1-vac in patients with newly diagnosed astrocytomas^20,21^. Notably, CD4^+^ T cell phenotyping following H3K27M-vac revealed no evidence of induction of immunosuppressive regulatory T cells. While the immunogenicity of the short peptide vaccine H3.3K27M_26-35_ is restricted to HLA-A*02^+^ patients^17^, we provide evidence of presentation of H3K27M neoepitope on MHC class II on tumor cells and on APCs across multiple HLA types (Fig. 4). While killing activity of H3K27M-specific CD8^+^ T cells against HLA-A*02^+^ tumor cells with endogenous H3.3K27M expression remains controversial^17–19^ we have recently demonstrated that CD4^+^ T cell interaction with peptide MHC class II in glioma-infiltrating myeloid cells is critical for the fitness of glioma-infiltrating CD8^+^ T cells^22^. Further analyses in a larger clinical trial cohort will assess the suitability of MHC class II expression and neoepitope presentation in DMG as predictive markers for response to H3K27M-vac.

Second, both H3K27M-vac and IDH1-vac target clonal driver mutations in proteins that are expressed in all cells of primary and recurrent tumors and are functionally relevant for tumor growth^2,23^. Lack of clonality, in contrast, may explain why a peptide vaccine against EGFRvIII and EGFRvIII-targeting CAR T cells have failed to yield clinical benefits in glioblastomas^24–27^. In the cohort presented here, we observed initial tumor regression in six of eight patients with clinical stabilization for more than 6 months in four patients. Lack of post-treatment biopsies precluded the analysis of possible immune evasion, for instance through lack of presentation.

Third, H3K27M-vac-induced T cell clones were detected in both peripheral blood and CSF and expanded concurrently to radiographic tumor regression. Although a conclusive assessment of the diversity of H3K27M-expanded TCRs is not possible with only a few clonotypes from a single patient, motif analysis showed sequence similarities of the CDR3β region. H3K27M-vac induced immune responses against H3K27M across different patient HLA class II types with a latency of up to 18 weeks irrespective of concomitant immune checkpoint blockade. Although sustained mutation-specific immune responses were detected in 80% of responding patients in our cohort, the strength of H3K27M-vac-induced peripheral immune responses tended to decrease over time and in one patient (ID 5) a previously existing ELISpot response was no longer detectable immediately before tumor progression. As a consequence, administration of H3K27M-vac to patients with newly diagnosed H3K27M^+^ DMG concomitant to standard-of-care first-line therapy could maximize its therapeutic benefit by allowing more time for CD4^+^ T cell-mediated antitumor immunity to become effective. An active multicenter, phase I clinical trial for adult patients with newly diagnosed H3K27M^+^ DMG integrates H3K27M-vac in combination with atezolizumab into standard-of-care radiotherapy (NCT04808245).

## Methods

### Patient selection and treatment schedule

Patients received H3K27M-vac between August 2017 and November 2022 at the University Hospitals of Heidelberg and Mannheim. Treatment was approved by the institutional review board and ethics committee. All patients provided written signed informed consent according to CARE guidelines and in compliance with the Declaration of Helsinki principles. Patients received no compensation for participation in this compassionate use program. Only adult patients of all sexes and genders with unequivocal disease progression of histologically confirmed H3K27M^+^ DMG were offered to receive treatment with H3K27M-vac. Sex was determined based on self-report. Concomitant anti-PD-1 therapy was allowed depending on the treating physician’s discretion. As anti-PD-1 therapy is not approved for the treatment of DMG in Germany, the exact anti-PD-1 drug was dependent on availability. K27M substitution was determined by immunohistochemistry, hence allowing no differentiation between mutations in H3F3A and HIST2H1B/C. Exclusion criteria included concomitant treatment with dexamethasone (or equivalent) >4 mg d^−1^, Karnofsky performance index (KPI) < 70 and age <18 years. All patients had received radiotherapy in combination with chemotherapy with TMZ before the start of therapy. Radiation doses and the number of chemotherapy cycles at a dose of 200 mg m^−2^ are specified for each patient in Extended Data Table 1. Treatment consisted of vaccinations with H3K27M-vac in weeks 0, 2, 4, 6, 10, 14, 18 and 22, but was stopped in case of PD. After week 22, patients with stable disease were offered to continue vaccinations every three months until PD. MRI assessment was conducted every 12 ± 2 weeks. Patients were assessed for AEs by CTCAE v.5.0 on every visit for vaccination and every 12 weeks thereafter. According to good clinical practice, an AE was defined as any untoward medical occurrence during treatment with H3K27M-vac irrespective of causal relationship. AEs were judged to be treatment-related if the relationship to treatment was ‘possible’, ‘probable’ or ‘definite’. Disease progression and events which are unequivocally related to disease progression regardless of their outcome were not considered AEs. One brief partial seizure and one brief generalized seizure were not associated with disease progression, but were judged to be disease related in analogy to most clinical trials in neuro-oncology. All remaining AEs that were considered disease related occurred less than a week before MRI that showed PD. Regimen-limiting toxicity was defined as an occurrence of any treatment-related AE >grade 2 during the treatment phase.

### H3K27M-vac treatment

H3K27M-vac consists of 300 μg H3K27M 27-mer peptide (p14-40, KAPRKQLATKAARMSAPSTGGVKKPHR) synthesized by the good manufacturing practice (GMP) facility of the University of Tübingen, Germany and was emulsified in Montanide (ISA50) by the GMP facility at the University Hospitals of Heidelberg and Mannheim, Germany at most 24 h before application as described elsewhere^20^. H3K27M-vac was injected subcutaneously into the abdominal skin or thigh using 20-gauge needles or 21-gauge needles. The place for the subsequent injections were as close as possible to the previous injection site for all vaccinations. Ideally, the same draining lymph node was targeted for all the vaccinations. In cases of unacceptable local site reactions to the vaccination or imiquimod, the injection sites were changed but were still as close as possible to the original injection site. In such a case, subsequent vaccinations were applied to this newly chosen vaccination site (Extended Data Fig. 1a). At 15 min after injection topical imiquimod (5%, Aldara; one sachet) was applied to an area of 5 × 5 cm around the site of injection of the vaccine and sealed with 5 × 5 cm of opsite flexifix (Smith&Nephew, product no. 7478029). Patients were instructed to leave Aldara on the skin for approximately 8 h and to wash the area where Aldara was applied with mild soap and water afterwards. At 24 h after vaccination patients applied another sachet of Aldara and washed the area approximately 8 h afterwards as described above. Labour LS s.e. & Co. in Germany performed quality controls for content, sterility and absence of endotoxin for each emulsion.

### Disease assessment

Clinical status was assessed during patient visits by a clinical neuro-oncologist. MRI assessment, including diagnosis of PsPD, applied the iRANO criteria on standardized MRIs that were obtained at least every 3 months. As in the NOA16 study^20^, PsPD was defined as an increase in the size of the tumor on T2-FLAIR MRI sequences and/or the new appearance or enlargement of contrast-enhancing lesions followed by stabilization or regression on follow-up MRI. Tumor sizes for Fig. 3a were determined by the product of maximal orthogonal diameters on T1-weighted contrast-enhanced MRI imaging and cerebral lesions were classified into measurable and non-measurable lesions based on iRANO criteria (cerebral lesion with both maximal orthogonal diameters >10 mm were classified as measurable).

### PBMC isolation

Heparinized blood from patients was diluted with phosphate-buffered saline (PBS) followed by density-gradient centrifugation (800 *g* without brake at room temperature) in Leucosep tubes (Greiner Bio-One) that contained Biocoll Separation Solution (Biochrom). Isolated PBMCs were subsequently frozen in 50% freezing medium A (60% X-Vivo20, 40% fetal calf serum (FCS)) and 50% medium B (80% FCS and 20% dimethylsulfoxide) and stored in liquid nitrogen at −140 °C until analysis.

### IFN-γ ELISpot assays of PBMCs

After hydrophilization with 35% ethanol, ELISpot HTS plates with white-bottom (Millipore, Merck, MSIPS4W10) were coated overnight at 4 °C with anti-human IFN-γ (1-D1K, Mabtech, 3420-3-250) and blocked with X-Vivo20 (Lonza) containing 1% BSA. PBMCs were thawed, rested in X-Vivo20 medium for 16 h, plated at 3 or 4 × 10^5^ cells per well as indicated and stimulated with 100 μl peptide solution at a concentration of 20 µg ml^−1^. Mutant H3K27M (p14-40, KAPRKQLATKAARMSAPSTGGVKKPHR), wild-type H3 (p14-40, KAPRKQLATKAARKSAPSTGGVKKPHR) or MOG (p35–55, MEVGWYRPPFSRVVHLYRNGK) at equal concentrations were used for stimulation. Aqua ad iniectabilia (Braun) with 10% dimethylsulfoxide (vehicle) at equal volume to peptide solution were used as negative controls and 1 μg staphylococcal enterotoxin B (Sigma-Aldrich) per well as well as 0.05 μg CMV with 0.05 μg AdV per well were used as positive controls. In selected experiments, 10 µg ml^−1^ MHC I (W6-32) or 90 µg ml^−1^ MHC II (Tü39) blocking antibodies were added to peptide-stimulated wells. After 40 h of incubation, biotinylated anti-human IFN-γ antibodies (7-B6-1, Mabtech, 3420-6-250), streptavidin-ALP (Mabtech, 3310-10-1000) and ALP color development buffer (Bio-Rad, 170-6432) were used for detection of IFN-γ-producing cells. An ImmunoSpot Analyzer (ImmunoSpot/CTL Europe) was used for quantification of spot counts. Measurements were performed in triplicate with rare exceptions where duplicates had to be used due to low cell numbers. T cell responses were defined as a significantly higher number of s.f.u. after stimulation with K27M-mutant H3 compared to wild-type H3, as assessed by a two-sided *t*-test with a false discovery rate of 5% as determined by a two-stage step-up method of Benjamini Krieger and Yekutieli, imposed for each patient individually.

### Flow cytometry

T cell cytokine secretion was measured using flow cytometry-based ICS ex vivo and after a 2-week in vitro restimulation. Briefly, PBMCs from a pre- or post-vaccination time point were thawed, rested overnight at 2–10 × 10^6^ cells ml^−1^ in cytokine-free X-Vivo20 (Lonza, BE04-380Q) and on the next day restimulated either immediately (ex vivo ICS, 1 × 10^6^ PBMCs per setup) or after in vitro expansion (0.4 × 10^6^ cells). PBMCs were stimulated for 6 h with H3K27M-mut or H3K27M-wt peptide at 20 µg ml^−1^. Unstimulated cells and PMA/ionomycin (0.05 µg ml^−1^ and 1 µg ml^−1^)-stimulated cells served as positive and negative controls, respectively. After 1 h of incubation, protein transport inhibitor brefeldin A (GolgiPlug, BD, 555029) was added to each well at a 1:1,000 dilution. At the end of restimulation, cells were collected, incubated with a live-dead discriminator (Fixable Viability Dye APC-R700 in PBS, Invitrogen, 564997) and stained with extracellular backbone antibodies (CD3-Fitc, clone HIT3a, BD, 561802), CD4-BV605 (clone SK3, BD, 565998), CD8-PerCP-Cy5.5 (clone RPA-T8, Invitrogen, 45-0088-42) and either CD45RA-APC-H7 (clone 5H9, BD, 561212), CCR7-BV711 (clone 150503, BD, 566602), PD-1-PE (clone EH12.1, BD, 560795) or CD25-BV605 (clone 2A3, BD, 562660) and HLA-DR-APC-H7 (clone G46-6, BD, 561358) in FACS buffer (PBS + 2% FCS, Biochrom). Subsequently, intracellular staining was performed with IFN-γ-BV421 (clone, 4S.B3, BD, 564791) and TNF-α-APC (clone, Mab11, BioLegend, 502912) and FoxP3-PE (clone 259/C7, BD, 560046) antibodies using Cytofix/Cytoperm reagents (BD Biosciences) according to the manufacturer’s instructions. Staining of expanded cells was limited to backbone antibodies and intracellular staining of IFN-γ and TNF-α. Staining was carried out at 4 °C protected from light. All antibodies used have been titrated to achieve optimal signal to noise rations. Cells were acquired on a BD FACS Lyric and analyzed using FlowJo analysis software v.10.8.1 (Extended Data Fig. 5).

### Proximity ligation assay

Baseline paraffin-embedded glioma tissue was used for PLA as described in Bunse et al.^28^ H3 wild-type glioblastoma tissue from the archives of neuropathology were obtained with approval by the institutional review boards (Ethikkommission) to serve as negative control for PLA staining. Nonlinear adjustment (gamma changes) was used for visualization. Immunofluorescence co-staining was performed using mouse monoclonal anti-human GFAP (1:2,000 dilution, Cell Signaling Technology, 3670), rabbit polyclonal anti-human IBA1 (1:100 dilution, Wako, 019-19741), and secondary antibodies used were donkey anti-mouse Alexa Fluor 488 and donkey anti-rabbit Alexa Fluor 488 (all 1:300 dilution, Molecular Probes, Invitrogen, A-21202 and A-21206). For segmentation of PLA spots and nuclei an in-house developed macro for the ImageJ platform was used. Background was subtracted using the rolling ball background subtraction, Gaussian blur was used for filtering and foci as well as nuclei were segmented using the Find Maxima tool.

### Next-generation HLA typing

The QIAamp DNA Blood Mini kit (QIAGEN) was used to isolate genomic DNA from PBMCs of patients. A total of 100 µl DNA solution with a concentration of at least 20 ng µl^−1^ was submitted at room temperature for high-resolution HLA typing to DKMS, Germany. Briefly, at DKMS, long-range PCRs were performed, amplicons were fragmented and used for next-generation sequencing on an Illumina MiSeq device. The full HLA class I gene and exons 2–5 of HLA class II genes were analyzed using the NGSengine (GenDx) software. Depending on the resolution, typing results were delivered either as G-code or MAC/NMDP-code.

### MHC II immunohistochemistry

Immunohistochemical analysis was carried out on 3-µm thick FFPE tissue sections affixed onto StarFrost Advanced Adhesive slides (Engelbrecht), followed by drying at 80 °C for 15 min. Immunohistochemistry was conducted using a BenchMark Ultra immunostainer (Ventana Medical Systems). The slides were pretreated with Cell Conditioning Solution CC1 (Ventana Medical Systems) for 32 min at room temperature. The primary antibody (MHC II, 1:100 dilution, clone CR3/43, DAKO, Agilent) was incubated at 37 °C for 32 min and then the Ventana standard signal amplification and UltraWash steps were performed. Counter-staining was carried out with hematoxylin for 4 min, followed by bluing reagent for 4 min. The visualization of the immunostaining was achieved using the UltraView Universal DAB Detection kit (Ventana Medical Systems). Scanning of the stained slides was accomplished using the Aperio AT2 Scanner (Aperio Technologies). QuPath (v.0.2.3) software was utilized for image analysis, which involved determining the total number of tumor cells within selected regions based on nuclear hematoxylin staining, as well as quantifying the total number of MHC II-positive cells in each image. The primary read out was determined by calculating the percentage of MHC II-positive cells/nuclei from each image.

### Peptide-based T cell expansion assay

PBMCs were expanded under exposure to mutant H3K27M (p14-40) peptide to enrich peptide-reactive T cell clones. Briefly, cells were thawed, transferred into X-Vivo20 (Lonza, BE04-380Q) medium supplemented with 2% AB serum (Sigma, H4522) and rested overnight as described above. On day 1, cell suspensions were adjusted to 1 × 10^6^ cells ml^−1^ and half of the available volume was plated at 500 µl per well into a 24-well plate. All remaining cells were plated at the same density in a second 24-well plate. Individual wells were pulsed with either (1) 4 µg ml^−1^ H3-mut (p14-40), (2) 4 µg ml^−1^ H3-wt (p14-40) or (3) no peptide to control for unspecific expansion. Both plates were placed in a 37 °C CO_2_ incubator. After 4 h, non-adherent cells of the plate that was not pulsed with peptide were plated on top of peptide-pulsed cells at a final concentration of 1 × 10^6^ cells ml^−1^ and per well.

Cultures were supplemented with cytokine-containing medium on day 4, 7, 9 and 11 by replacing half of the medium per well (final cytokine concentrations per well were 50 IU ml^−1^ interleukin (IL)-2 (Novartis), 25 ng ml^−1^ IL-7 (Miltenyi, 130-095-367) and 25 ng ml^−1^ IL-15 (Miltenyi, 130-095-760)). On day 13–15, cells were transferred into cytokine-free medium and on the following day, peptide-specific expansion of T cells was verified by IFN-γ ELISpot or ICS. IFN-γ ELISpot assays were performed as described previously^20^. Briefly, cells were plated at a density of 5 × 10^4^ cells per well and restimulated with 10 µg ml^−1^ mutant H3K27M (p14-40) peptide, 10 µg ml^−1^ wild-type H3 (p14-40) peptide, left unstimulated as a negative control or exposed to PMA/ionomycin (0.02 µg ml^−1^ and 1 µg ml^−1^) as a positive control. The assay was stopped after 44 h and spots were quantified using an ImmunoSpot Analyzer (Cellular Technology).

### TCRβ deep sequencing

Genomic DNA from tissue, blood or CSF of patient ID 1 was isolated using the DNeasy Blood and Tissue kit (QIAGEN, 69504). Libraries for TCR β-chain deep sequencing were prepared using the hsTCRB kit V4b (Adaptive Biotechnologies) according to the manufacturer’s protocol and sequenced on an Illumina MiSeq device. Sequencing was performed by the Genomics & Proteomics Core Facility (German Cancer Research Center). Data were processed (demultiplexing, trimming, gene mapping) using the immunoSEQ platform from Adaptive Biotechnologies. Motif analysis was carried out using the XSTREME Tool^29^ after removing the recurring CAS sequence from all top ten TCRs from TCRβ deep sequencing. Shuffled input sequences were used as control sequences.

### Statistical analysis

All statistical analyses were carried out in R v.3.6.1 and used a significance level of 5%. Association of patient characteristics with H3K27M-specific immune responses were assessed by Fisher’s exact test. The R software packages used to calculate statistics and to illustrate the data were grid_3.6.1, stats_3.6.1, graphics_3.6.1, grDevices_3.6.1, utils_3.6.1, datasets_3.6.1, methods_3.6.1, base_3.6.1, reshape2_1.4.3, survival_3.2-13, survminer_0.4.9, ggpubr_0.2.5, magrittr_2.0.3, ggplot2_3.3.2, swimplot_1.2.0, circlize_0.4.9, RColorBrewer_1.1-2, ComplexHeatmap_2.5.1 and openxlsx_4.1.4.

### Reporting summary

Further information on research design is available in the Nature Portfolio Reporting Summary linked to this article.

## Online content

Any methods, additional references, Nature Portfolio reporting summaries, source data, extended data, supplementary information, acknowledgements, peer review information; details of author contributions and competing interests; and statements of data and code availability are available at 10.1038/s41591-023-02555-6.

### Supplementary information

Supplementary Information Supplementary Figs. 1–14.

Reporting Summary

### Source data

Source Data Fig. 1 Statistical source data for Figs. 1a,b, 2b,c, 3a,b and 4e and Extended Data Figs. 1, 2a–c, 3a,b, 4h and 5a,f,k,l.

## Figures and Tables

**Extended Data Table 1:**
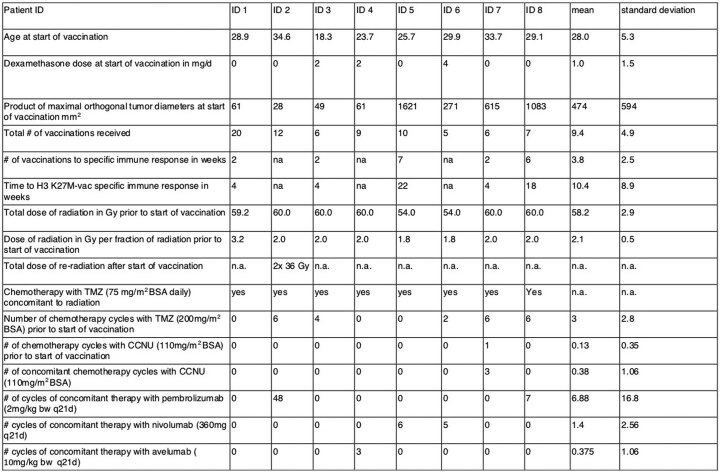
Quantitative patient characteristics

**Extended Data Table 2:**
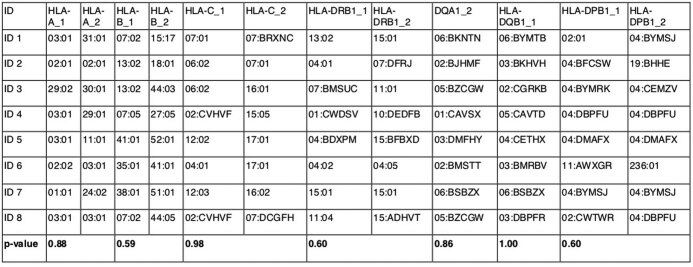
HLA types of patients, p-values from fisher exact test of association of HLA type and H3K27M-specific immune response

G-codes are listed if typization exactly matched one G-code. For cases with shorter allele-strings (that is less allelels than G-code) or for cases with longer allele-strings (that is more possible results) NMDP-code is listed. Two.sided t-Test statistics was used.

## Data Availability

The primary data that support the findings of this study are not openly available due to patient privacy. Access can be granted by contacting K.S. (k.sahm@dkfz.de) and requires a data-access agreement; requests will be replied to within 4 weeks. All primary data are stored on the controlled access repository of the University Hospital Mannheim. Referenced datasets were not used in the study. Source data are provided with this paper.
