# Supplementary material for: A H3K27M-targeted vaccine in adults with diffuse midline glioma
Source: Nat Med. 2023 Sep 21;29(10):2586–92. doi: 10.1038/s41591-023-02555-6 (PMC10579055; doi:10.1038/s41591-023-02555-6)
Supplement: Supplementary file 1 — Supplementary Figs. 1–14. [file 41591_2023_2555_MOESM1_ESM.pdf]

nature medicine

Article https://doi.org/10.1038/s41591-023-02555-6

A H3K27M-targeted vaccine in adults with diffuse midline glioma

In the format provided by the authors and unedited

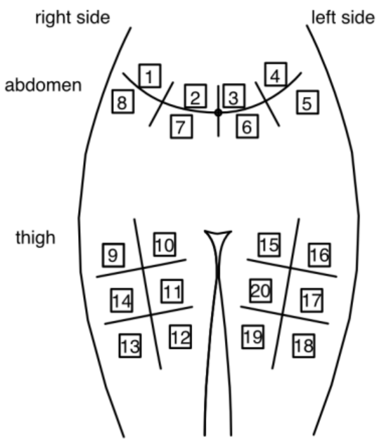

Supplementary Figure 1: Administration of H3K27M-vac
H3K27M-vac was injected subcutaneously in the lower abdomen or thigh followed by topical application of Imiquimod (5%, Aldara®) that was sealed by 5 x 5 cm of opsite flexifix.

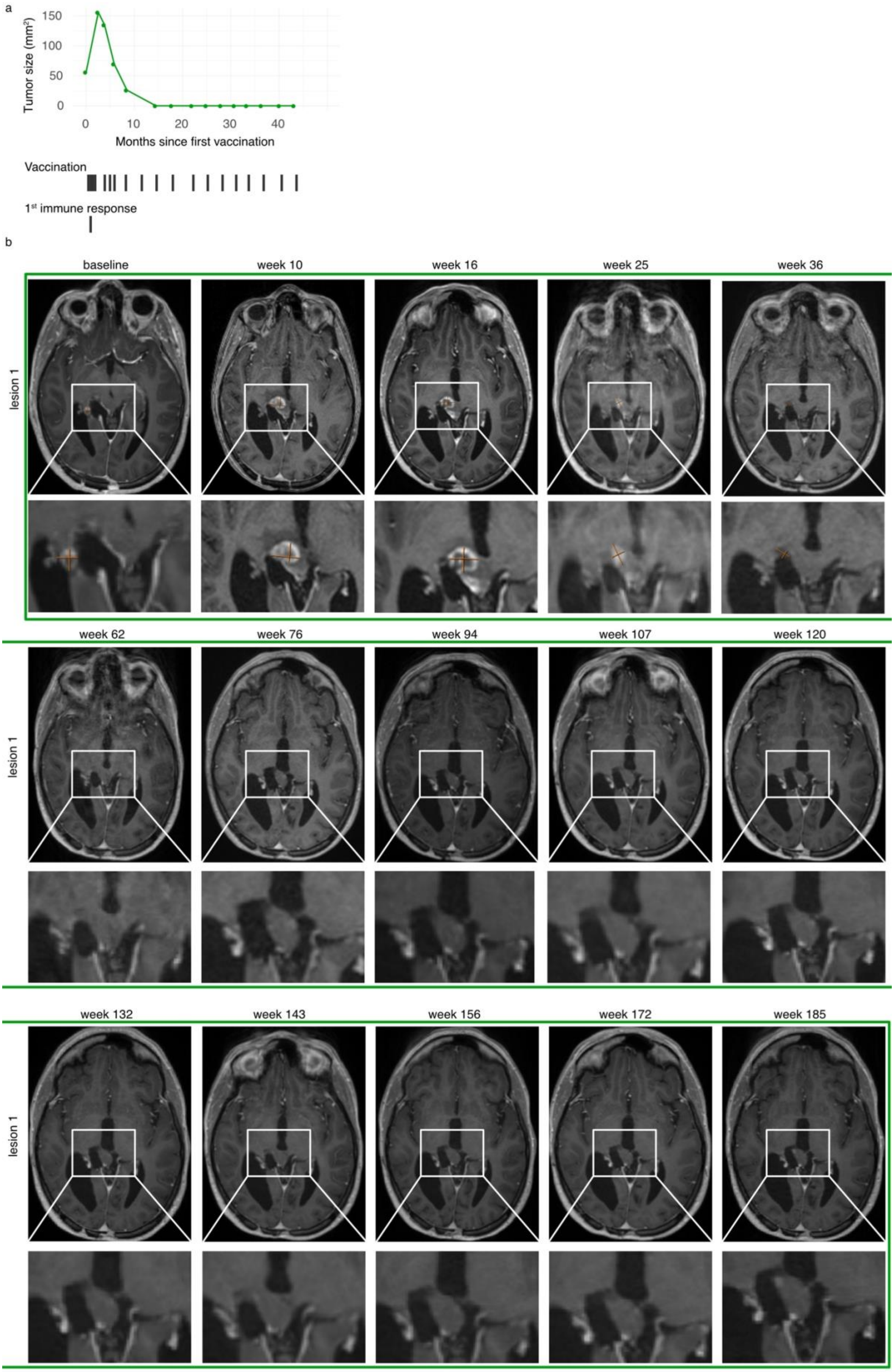

Supplementary Figure 2: Longitudinal course of T1 weighted contrast enhanced MRI images of patient ID 1

a, Tumor size in mm^2 (top), therapy in the observation period and first immune response (bottom) as a function of time in months from start of vaccination. Size determined by product of maximal orthogonal diameters on T1 weighted contrast enhanced MRI imaging. **b,** Corresponding axial MRI sequences with maximal orthogonal diameters of contrast enhancing lesion and corresponding measurements indicated in orange, if applicable. Green box indicates MRIs while ID 1 was on treatment with H3K27M-vac.

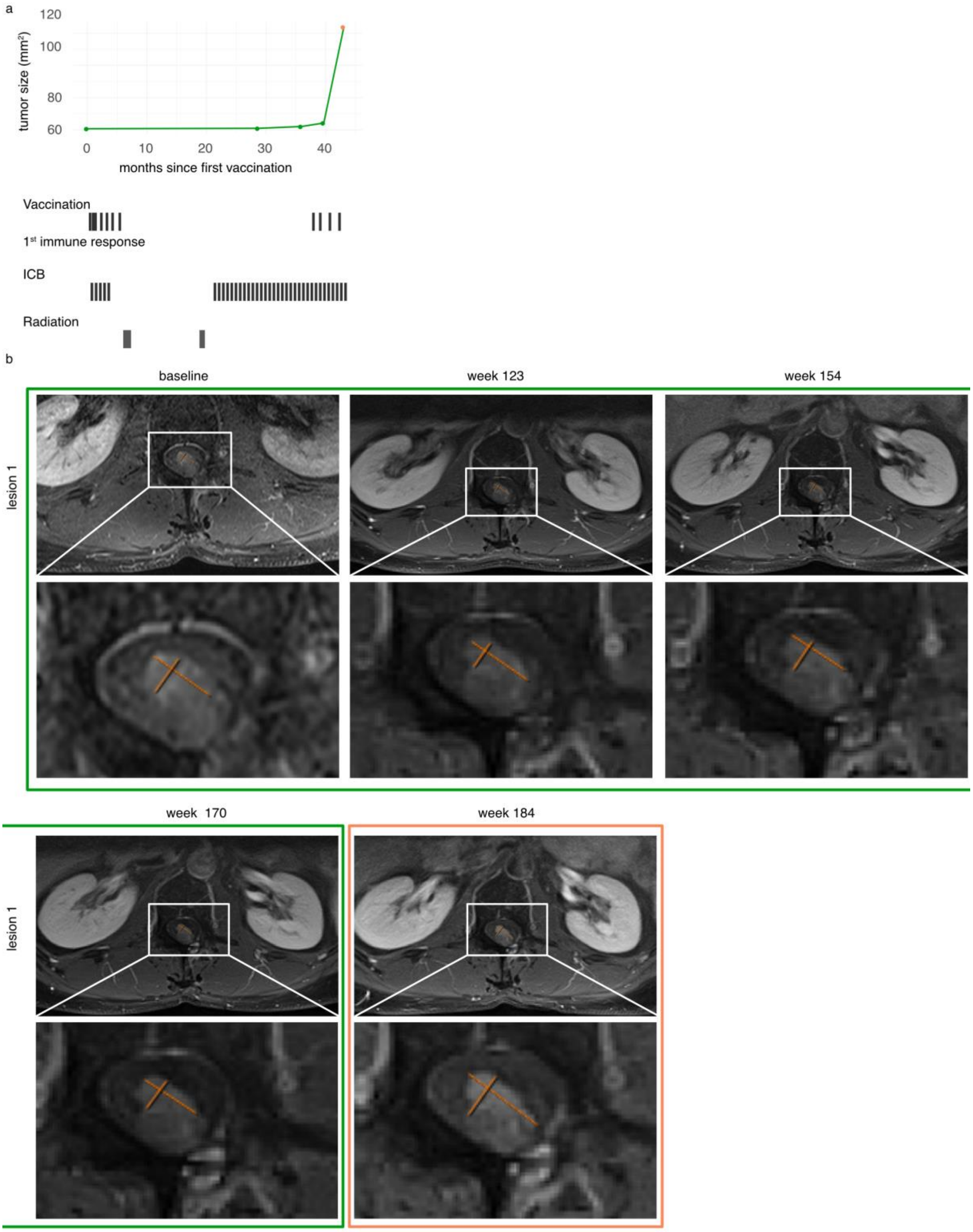

Supplementary Figure 3: Longitudinal course of T1 weighted contrast enhanced MRI images of patient ID 2
a, Tumor size in mm^2 (top), concomitant therapy (bottom) consisting of two re-irradiations and anti-PD1 therapy as a function of time in months from start of vaccination. No immune response to H3K27M peptide was detected in this patient throughout the observation period. Size determined by product of maximal orthogonal diameters on T1 weighted contrast enhanced MRI imaging. **b,** Corresponding axial MRI sequences with maximal orthogonal diameters of contrast enhancing lesion and corresponding measurements indicated in orange. Green box indicates MRIs while ID 2 was on treatment with H3K27M-vac, orange box signifies MRI with progressive disease marking the end of the observation period and end of treatment period with H3K27-vac.

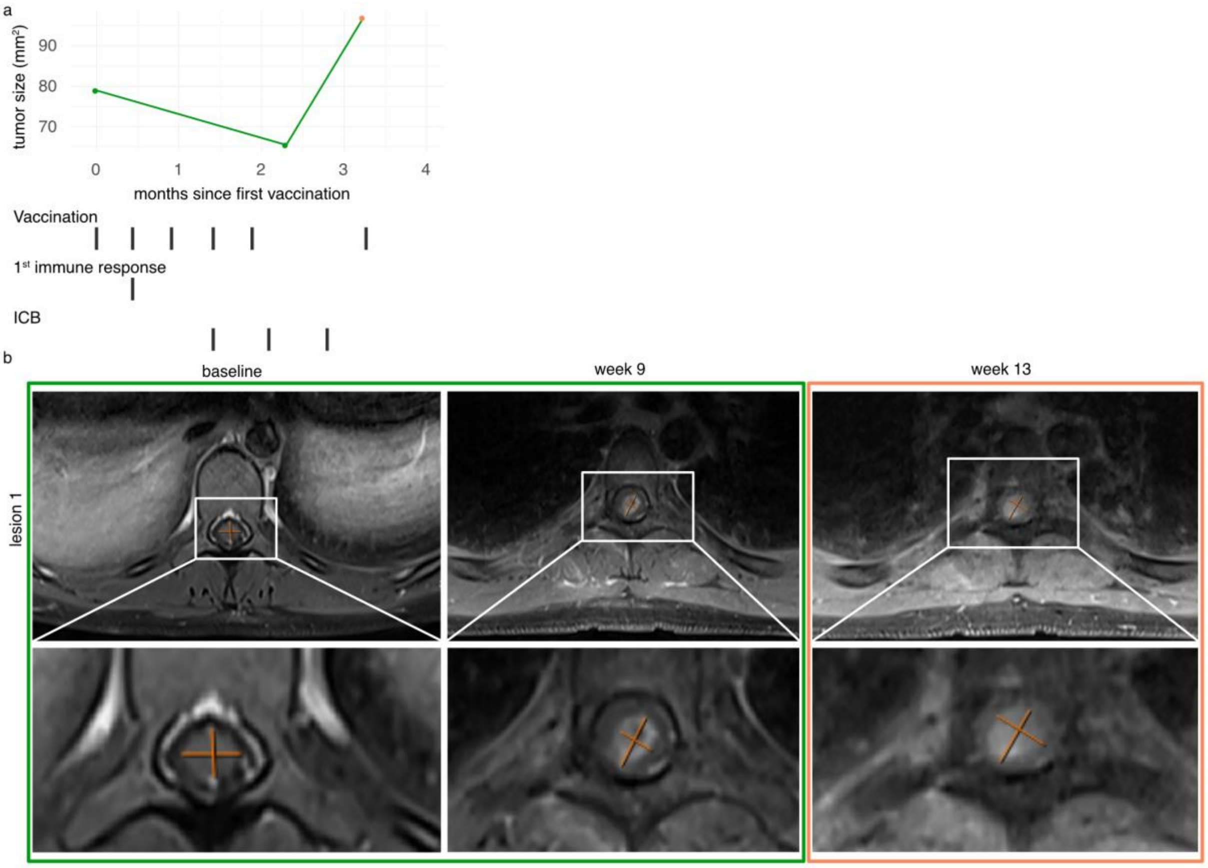

Supplementary Figure 4: Longitudinal course of T1 weighted contrast enhanced MRI images of patient ID 3
a, Tumor size in mm^2 (top) concomitant therapy (bottom) consisting of anti-PD1 treatment as a function of time in months from start of vaccination. Size determined by product of maximal orthogonal diameters on T1 weighted contrast enhanced MRI imaging. **b,** Corresponding axial MRI sequences with maximal orthogonal diameters of contrast enhancing lesion and corresponding measurements indicated in orange, if applicable. Green box indicates MRIs while ID 3 was on treatment with H3K27M-vac, orange box signifies MRI with progressive disease marking the end of the observation period and end of treatment period with H3K27-vac.

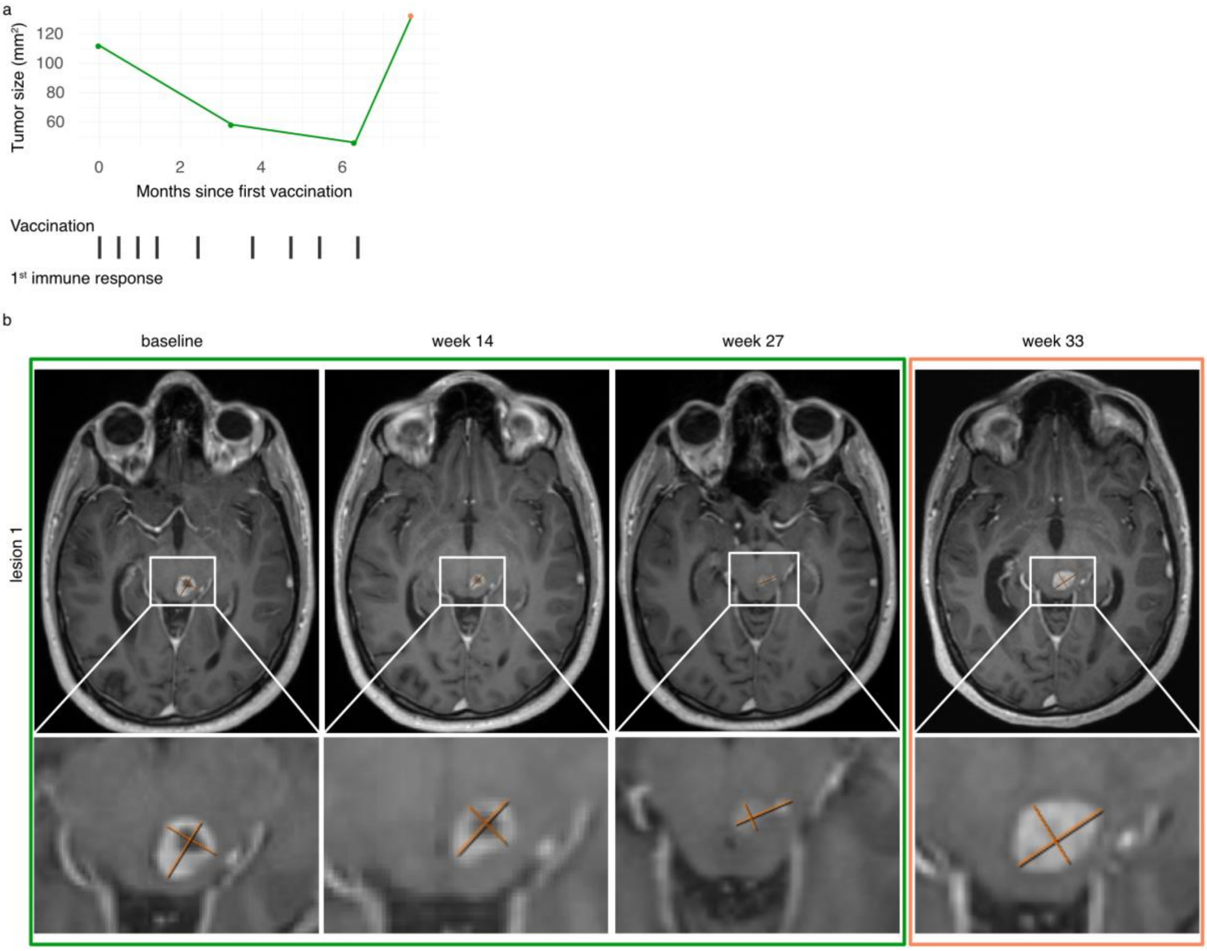

Supplementary Figure 5: Longitudinal course of T1 weighted contrast enhanced MRI images of patient ID 4
a, Tumor size in mm^2 (top) and therapy (bottom) in the observation period as a function of time in months from start of vaccination. No immune response to H3 K27M peptide was detected in this patient throughout the observation period. Size determined by product of maximal orthogonal diameters on T1 weighted contrast enhanced MRI imaging. **b,** Corresponding axial MRI sequences with maximal orthogonal diameters of contrast enhancing lesion and corresponding measurements indicated in orange. Green box indicates MRIs while ID 4 was on treatment with H3K27M-vac, orange box signifies MRI with progressive disease marking the end of the observation period and end of treatment period with H3K27-vac.

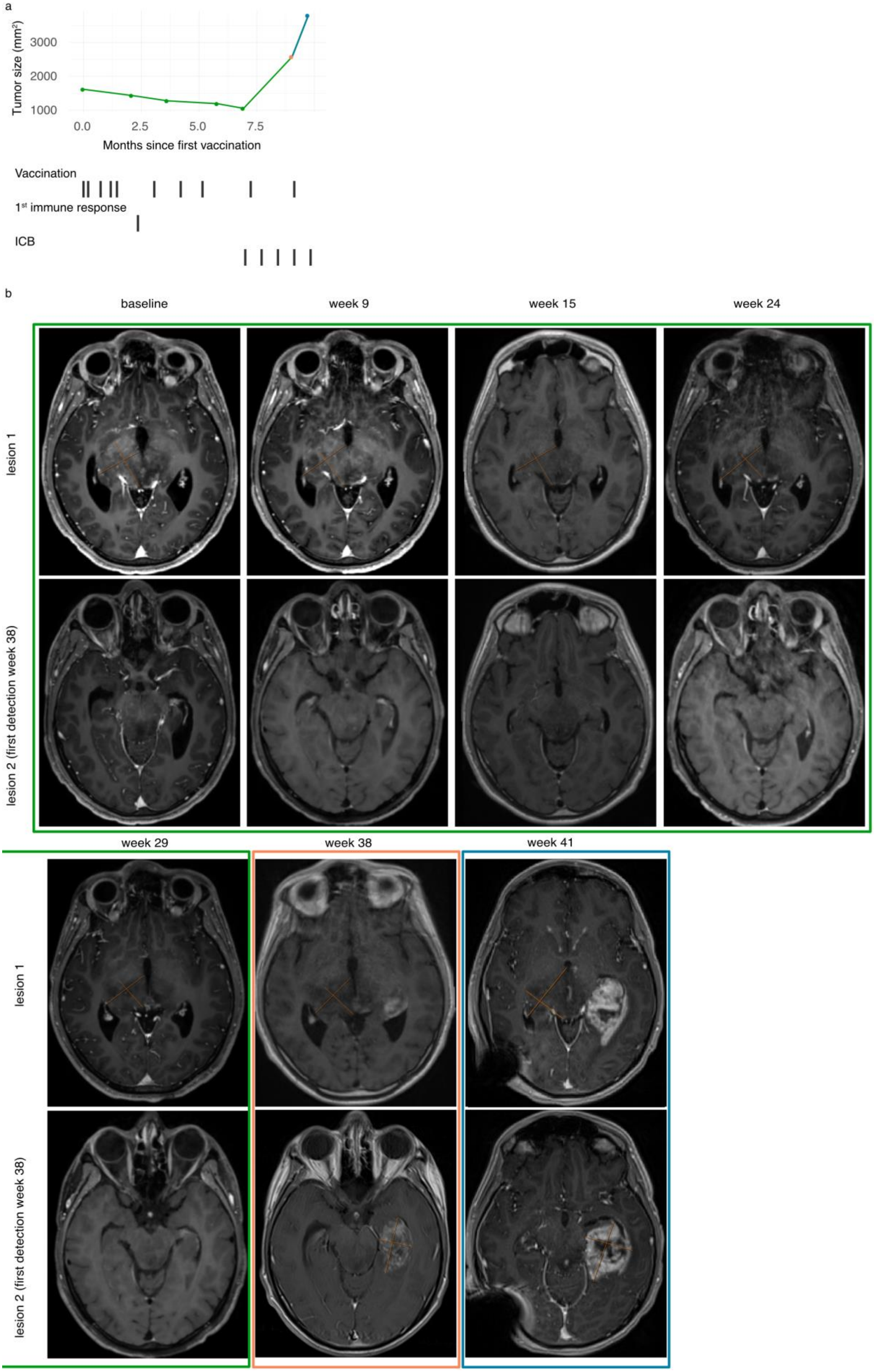
a
Tumor size (mm²)
3000
2000
1000
0.0
2.5
5.0
7.5
Months since first vaccination
Vaccination
1st immune response
ICB
b
baseline
week 9
week 15
week 24
lesion 1
lesion 2 (first detection week 38)
week 29
week 38
week 41
lesion 1
lesion 2 (first detection week 38)

Supplementary Figure 6: Longitudinal course of T1 weighted contrast enhanced MRI images of patient ID 5

a, Tumor size in mm^2 (top) concomitant therapy (bottom) consisting of anti-PD1 treatment as a function of time in months from start of vaccination. Size determined by product of maximal orthogonal diameters on T1 weighted contrast enhanced MRI imaging. **b,** Corresponding axial MRI sequences with maximal orthogonal diameters of contrast enhancing lesion and corresponding measurements indicated in orange, if applicable. A second, new lesion with contrast enhancement was detected upon week 38. Green box indicates MRIs while ID 5 was on treatment with H3K27M-vac, orange box signifies MRI with progressive disease marking the end of treatment period with H3K27-vac. The blue box marks an MRI that was obtained after the end of the treatment period.

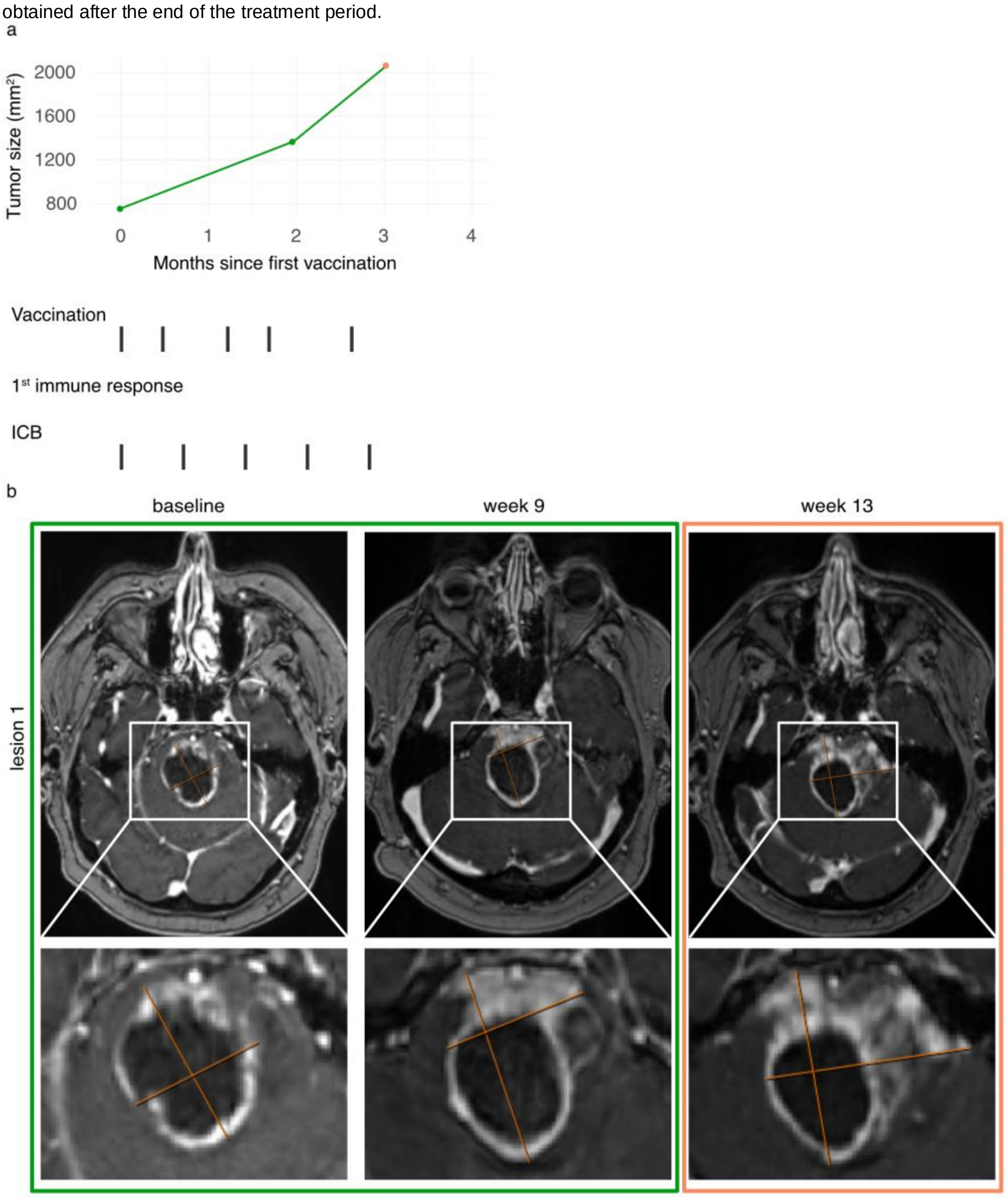

Supplementary Figure 7: Longitudinal course of T1 weighted contrast enhanced MRI images of patient ID 6

a, Tumor size in mm^2 (top) and concomitant therapy (bottom) consisting of of anti-PD1 treatment as a function of time in months from start of vaccination. No immune response to H3 K27M peptide was detected in this patient throughout the observation period. Size determined by product of maximal orthogonal diameters on T1 weighted contrast enhanced MRI imaging. **b,** Corresponding axial MRI sequences with maximal orthogonal diameters of contrast enhancing lesion and corresponding measurements indicated in orange. Green box indicates MRIs while ID 6 was on treatment with H3K27M-vac, orange box signifies MRI with progressive disease marking the end of the observation period and end of treatment period with H3K27-vac.

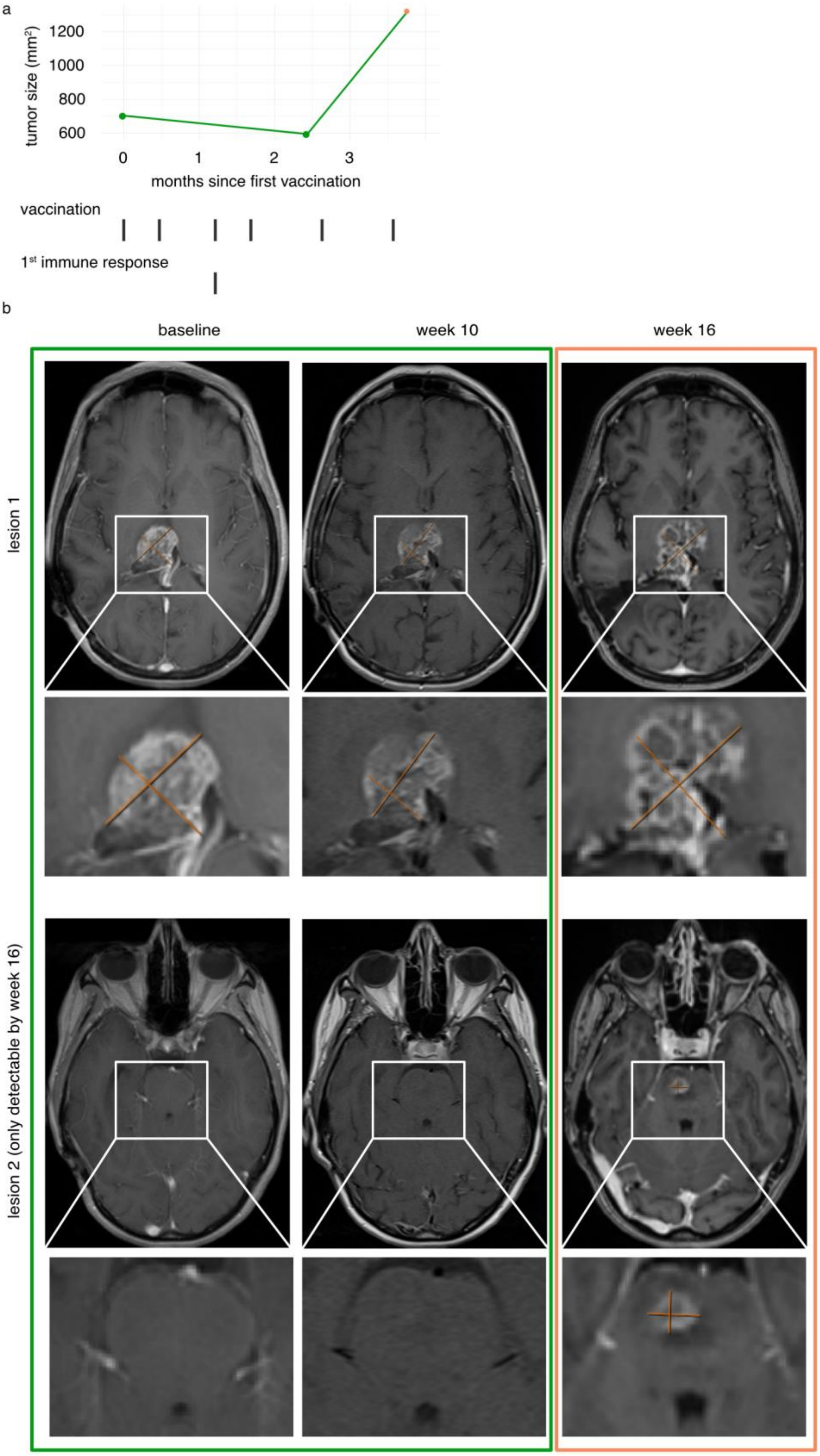

Supplementary Figure 8: Longitudinal course of T1 weighted contrast enhanced MRI images of patient ID 7
a, Tumor size in mm^2 (top) concomitant therapy (bottom) consisting of anti-PD1 treatment as a function of time in months from start of vaccination. Size determined by product of maximal orthogonal diameters on T1 weighted contrast enhanced MRI imaging. **b,** Corresponding axial MRI sequences with maximal orthogonal diameters of contrast enhancing lesion and corresponding measurements indicated in orange, if applicable. A second, new lesion with contrast enhancement was detected by week 16 for the first time. Green box indicates MRIs while ID 7 was on treatment with H3K27M-vac, orange box signifies MRI with progressive disease marking the end of the observation period and end of treatment period with H3K27-vac.

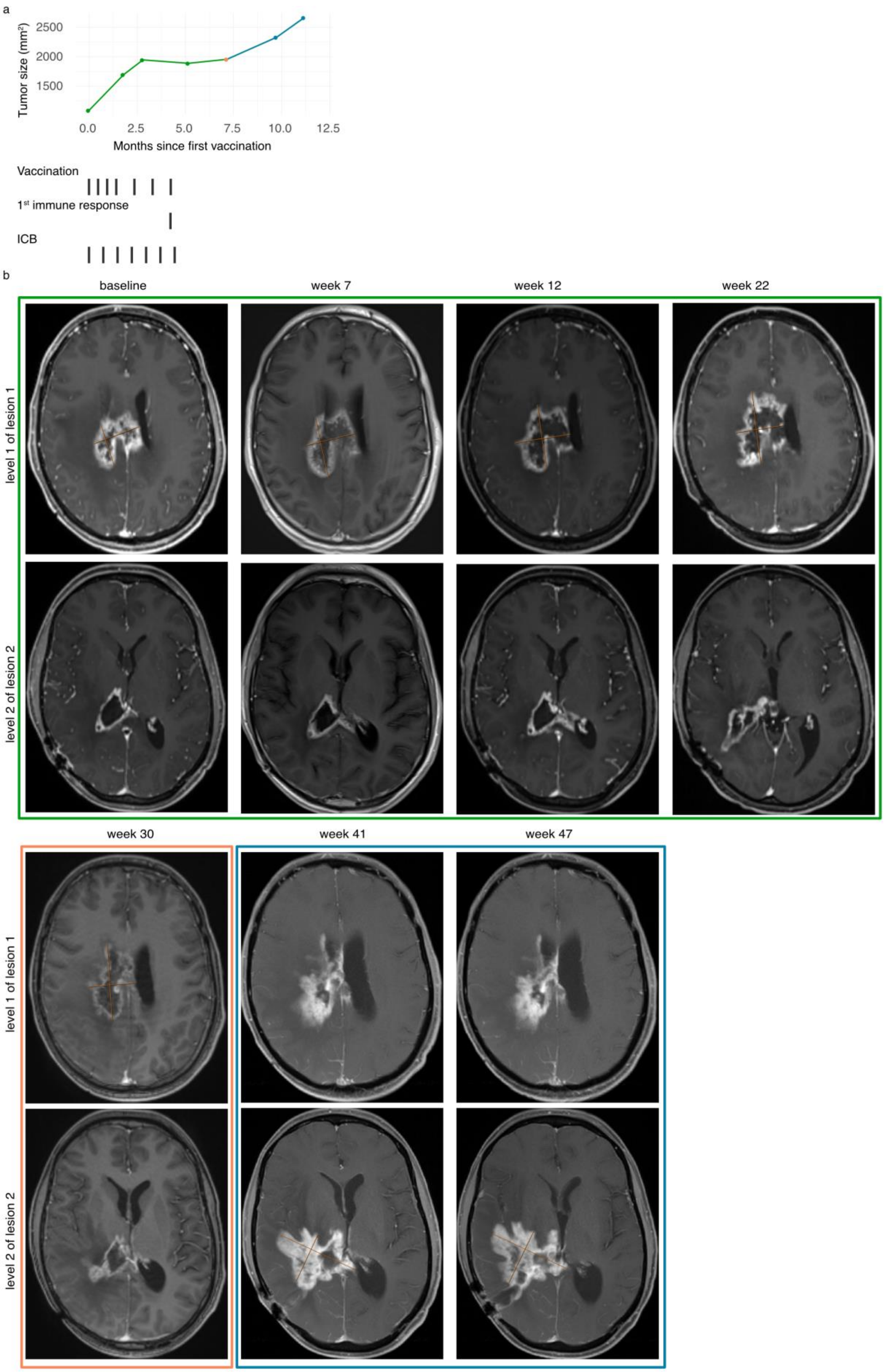

Supplementary Figure 9: Longitudinal course of T1 weighted contrast enhanced MRI images of patient ID 8

a, Tumor size in mm^2 (top) concomitant therapy (bottom) consisting of anti-PD1 treatment as a function of time in months from start of vaccination. Size determined by product of maximal orthogonal diameters on T1 weighted contrast enhanced MRI imaging. **b,** Corresponding axial MRI sequences with maximal orthogonal diameters of contrast enhancing lesion and corresponding measurements indicated in orange. While initially the maximal tumor diameter on axial MRI sequences was on level 1, by week 30 the same lesion grew on level 2 and had the maximal axial tumor diameter by week 41. Green box indicates MRIs while ID 8 was on treatment with H3K27M-vac, orange box signifies MRI with progressive disease marking the end of the treatment period with H3K27-vac. The blue box marks an MRIs that were obtained after the end of the treatment period.

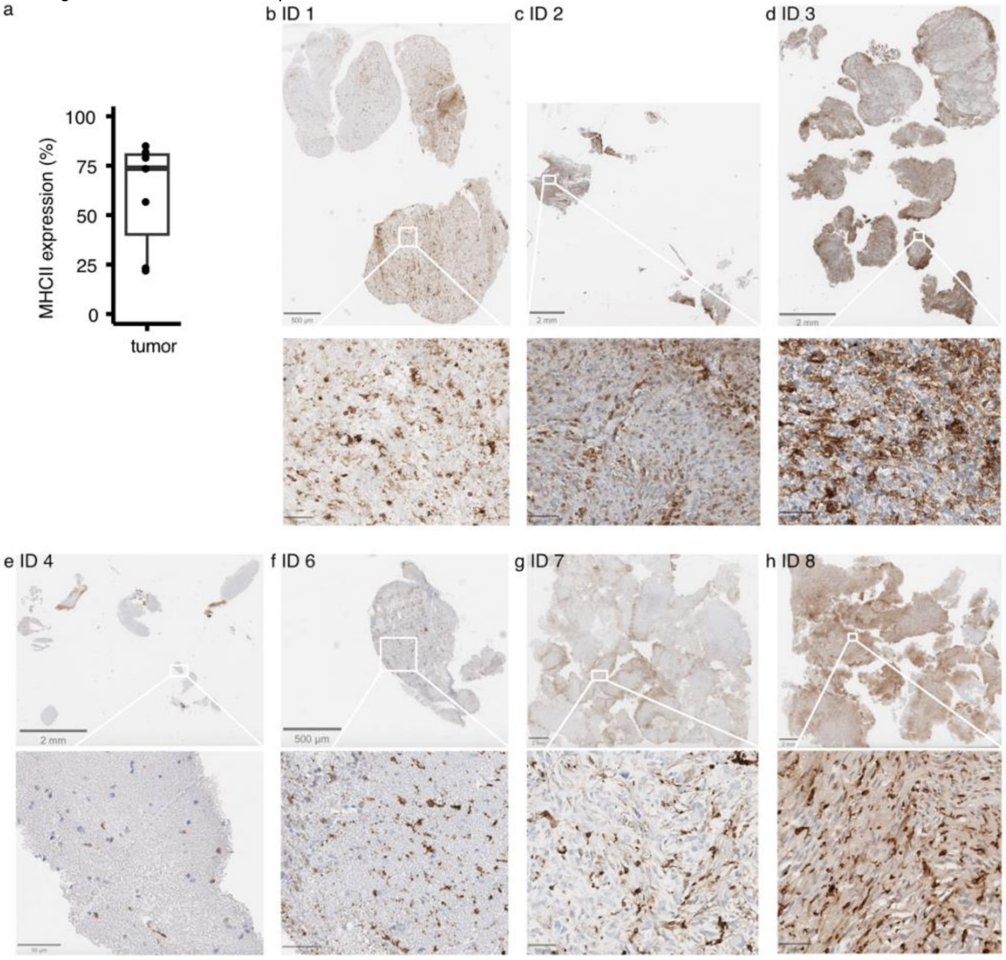

Supplementary Figure 10: HLA-DR immunohistochemistry of primary tumor tissue

a, Distribution of HLA-DR expression across all 7 patients with sufficient FFPE tissue (n=7 biologically independent patient samples). Box displays median and interquartile range (IQR), whiskers extend to the largest/smallest value no further than 1.5x IQR from the hinge. **b-h**: Immunohistochemistry (IHC) of HLA-DR expression across respective patients showing entire slide (top) and magnification (bottom). IHC was done once for every patient. Scale bars in top images in b and f = 500µm, in c-e and g,h = 10 µm, scale bar in magnifications (bottom) = 50µm.

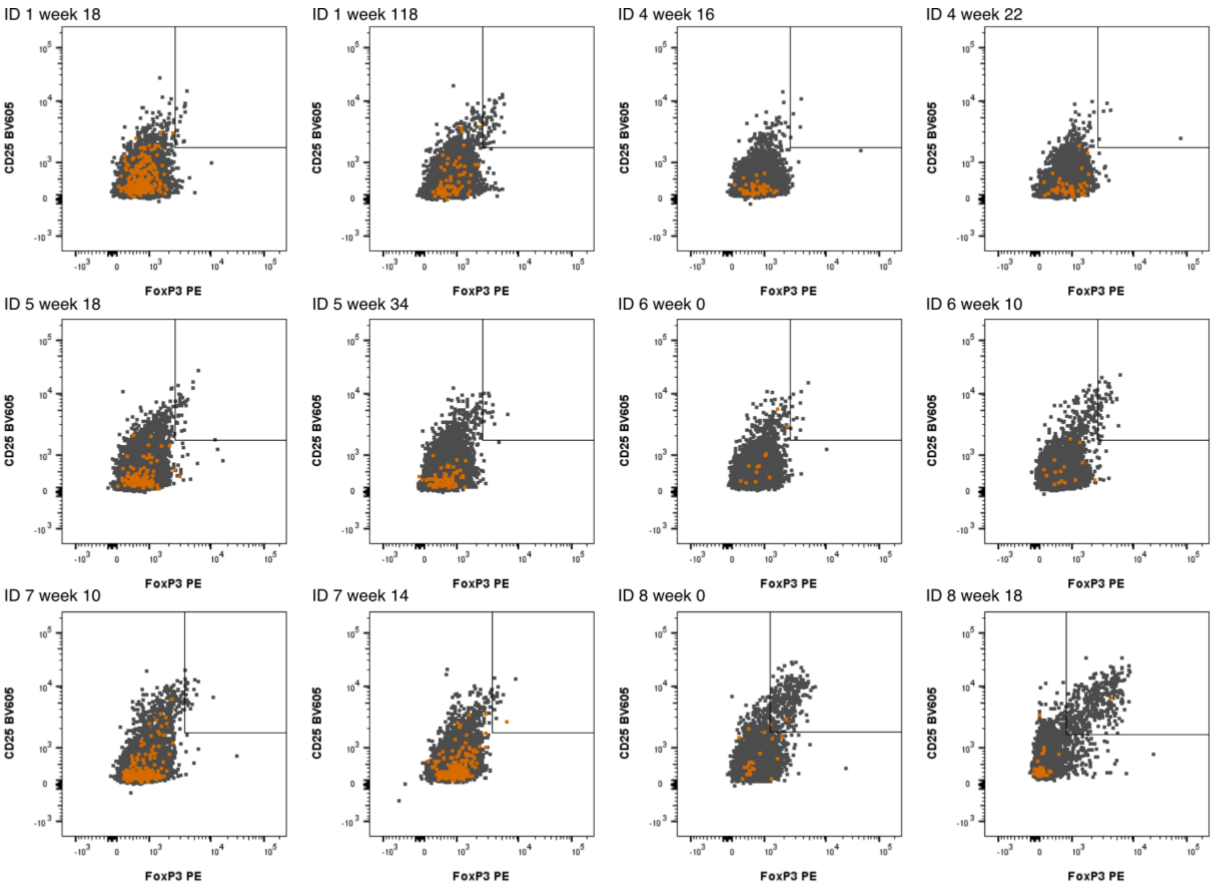

Supplementary Figure 11: Regulatory T-cells among H3K27M-reactive CD4⁺ T cells
Tumor Necrosis Factor-α (TNFα) expressing CD4⁺ T cells in orange among H3K27M-peptide expanded PBMC re-stimulated with H3-mut, gated on CD25 and FoxP3, demonstrating, that CD25⁺ FoxP3⁺ cells are not among the TNFα expressing CD4⁺ T cells. Patients and timepoints are indicated on the top left of each panel.

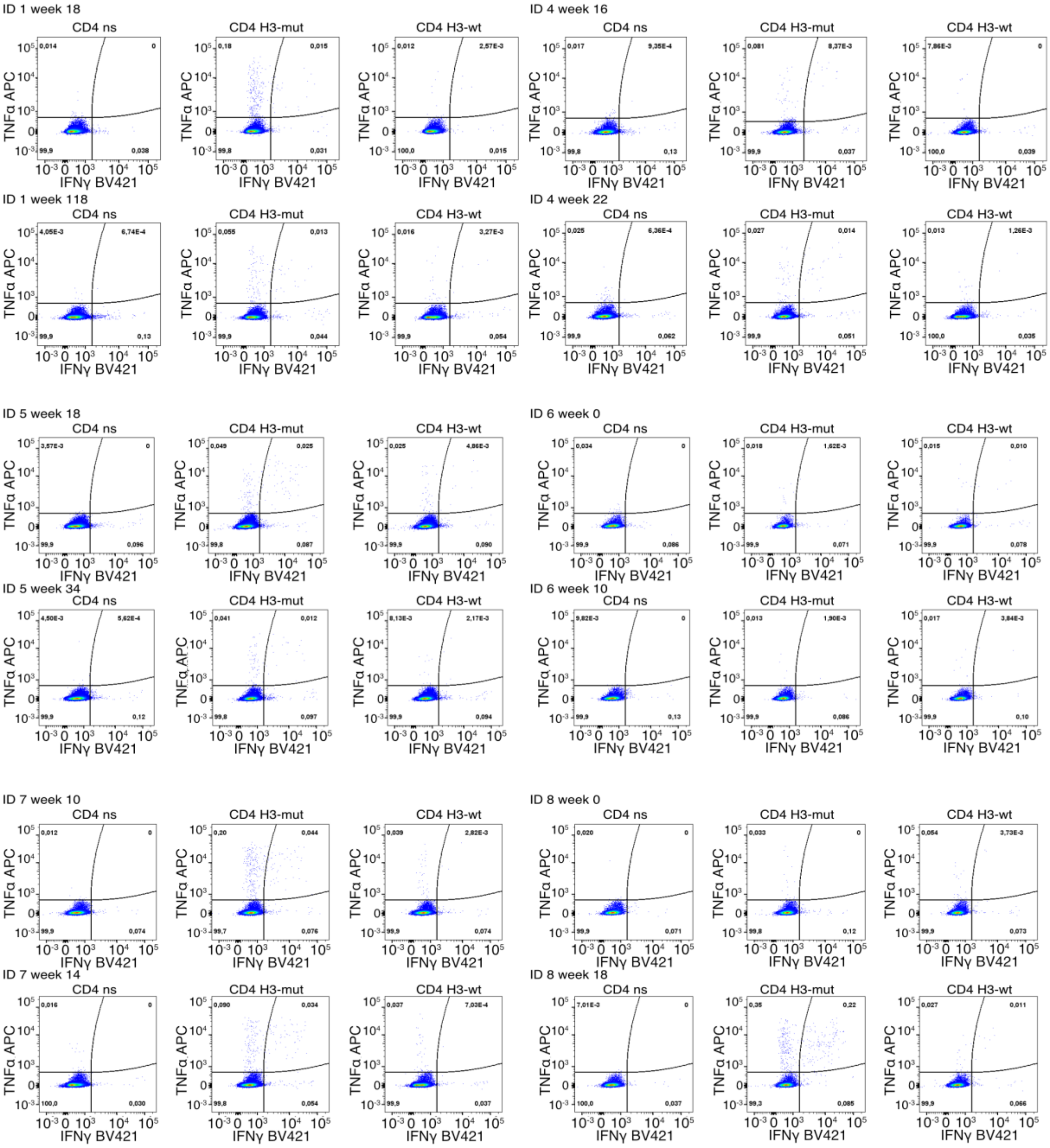

Supplementary Figure 12: FACS raw data for Figure 5f of PBMCs analyzed by ex vivo ICS.
Flow cytometry-based intracellular IFNγ and Tumor Necrosis Factor-α (TNFα) detection in H3K27M-peptide expanded PBMC after short-term (5h) re-stimulatiion with H3-wt or H3-mut or non-stimulated (ns), gated on living $CD3^+$ $CD4^+$ T cells.

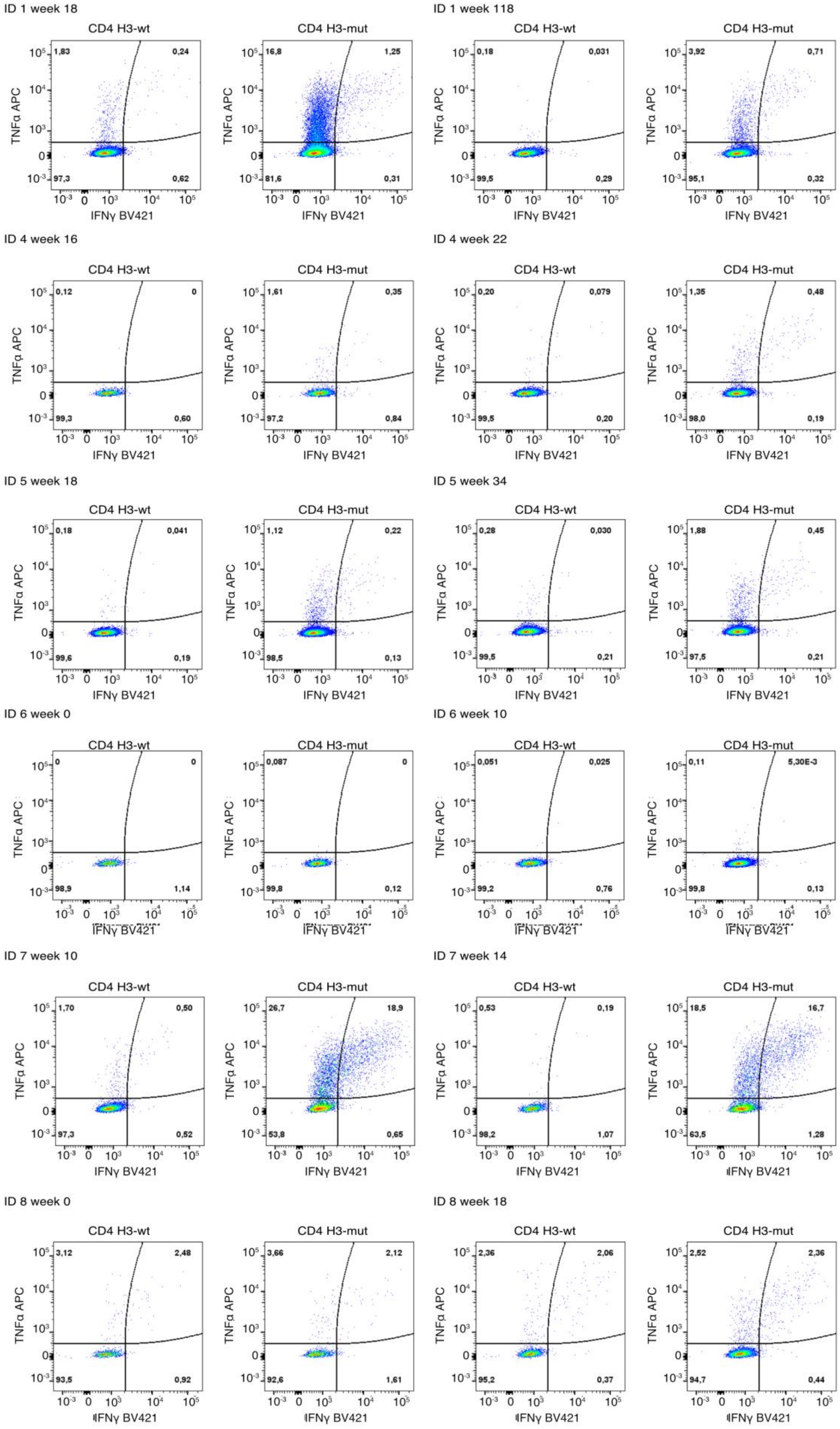

ID 1 week 18
CD4 H3-wt 1,83 0,24 97,3 0,62
CD4 H3-mut 16,8 1,25 81,6 0,31
ID 1 week 118
CD4 H3-wt 0,18 0,031 99,5 0,29
CD4 H3-mut 3,92 0,71 95,1 0,32
ID 4 week 16
CD4 H3-wt 0,12 0 99,3 0,60
CD4 H3-mut 1,61 0,35 97,2 0,84
ID 4 week 22
CD4 H3-wt 0,20 0,079 99,5 0,20
CD4 H3-mut 1,35 0,48 98,0 0,19
ID 5 week 18
CD4 H3-wt 0,18 0,041 99,6 0,19
CD4 H3-mut 1,12 0,22 98,5 0,13
ID 5 week 34
CD4 H3-wt 0,28 0,030 99,5 0,21
CD4 H3-mut 1,88 0,45 97,5 0,21
ID 6 week 0
CD4 H3-wt 0 0 98,9 1,14
CD4 H3-mut 0,087 0 99,8 0,12
ID 6 week 10
CD4 H3-wt 0,051 0,025 99,2 0,76
CD4 H3-mut 0,11 5,30E-3 99,8 0,13
ID 7 week 10
CD4 H3-wt 1,70 0,50 97,3 0,52
CD4 H3-mut 26,7 18,9 53,8 0,65
ID 7 week 14
CD4 H3-wt 0,53 0,19 98,2 1,07
CD4 H3-mut 18,5 16,7 63,5 1,28
ID 8 week 0
CD4 H3-wt 3,12 2,48 93,5 0,92
CD4 H3-mut 3,66 2,12 92,6 1,61
ID 8 week 18
CD4 H3-wt 2,36 2,06 95,2 0,37
CD4 H3-mut 2,52 2,36 94,7 0,44
TNFα APC
IFNγ BV421

Supplementary Figure 13: FACS raw data for Figure 5f of ex vivo peptide expanded PBMCs
Flow cytometry-based intracellular IFNγ and Tumor Necrosis Factor-α (TNFα) detection in PBMC with H3K27M-peptide expansion for two weeks, re-stimulated with H3-wt or H3-mut, gated on living $CD3^{+}$ $CD4^{+}$ T cell subsets.

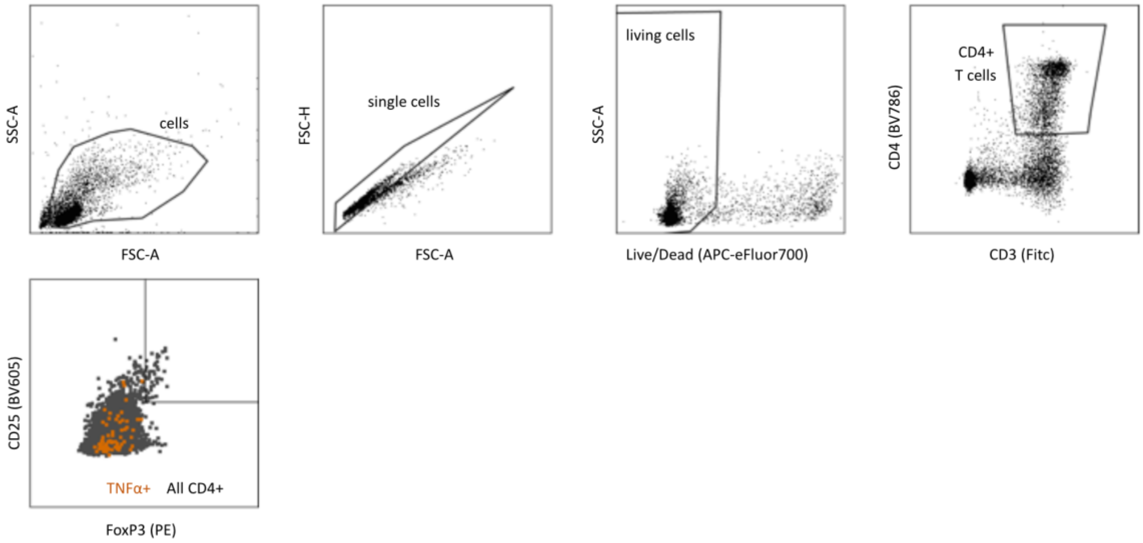

Supplementary Figure 14: Hierarchical gating as gating strategy
Exclusions of Debris (FSC-A vs SSC-A)/Exclusion of doublets (FSC-H vs FSC-A), Exclusion of dead-cells (SSC-A vs dead-cell-stain)/Definition of CD4 T cells (BV786 vs CD3-Fitc+).
