# Supplementary material for: A H3K27M-targeted vaccine in adults with diffuse midline glioma
Source: Nat Med. 2023 Sep 21;29(10):2586–92. doi: 10.1038/s41591-023-02555-6 (PMC10579055; doi:10.1038/s41591-023-02555-6)
Supplement: Supplementary file 2 — Reporting Summary [file 41591_2023_2555_MOESM2_ESM.pdf]

nature portfolio

Corresponding author(s): Katharina Sahm

Last updated by author(s): Aug 18, 2023

Reporting Summary

Nature Portfolio wishes to improve the reproducibility of the work that we publish. This form provides structure for consistency and transparency in reporting. For further information on Nature Portfolio policies, see our Editorial Policies and the Editorial Policy Checklist.

Statistics

For all statistical analyses, confirm that the following items are present in the figure legend, table legend, main text, or Methods section.

n/a	Confirmed	
☐	☒	The exact sample size (*n*) for each experimental group/condition, given as a discrete number and unit of measurement
☐	☒	A statement on whether measurements were taken from distinct samples or whether the same sample was measured repeatedly
☐	☒	The statistical test(s) used AND whether they are one- or two-sided<br>*Only common tests should be described solely by name; describe more complex techniques in the Methods section.*
☐	☒	A description of all covariates tested
☐	☒	A description of any assumptions or corrections, such as tests of normality and adjustment for multiple comparisons
☐	☒	A full description of the statistical parameters including central tendency (e.g. means) or other basic estimates (e.g. regression coefficient) AND variation (e.g. standard deviation) or associated estimates of uncertainty (e.g. confidence intervals)
☐	☒	For null hypothesis testing, the test statistic (e.g. *F*, *t*, *r*) with confidence intervals, effect sizes, degrees of freedom and *P* value noted<br>*Give P values as exact values whenever suitable.*
☒	☐	For Bayesian analysis, information on the choice of priors and Markov chain Monte Carlo settings
☒	☐	For hierarchical and complex designs, identification of the appropriate level for tests and full reporting of outcomes
☐	☒	Estimates of effect sizes (e.g. Cohen's *d*, Pearson's *r*), indicating how they were calculated

Our web collection on statistics for biologists contains articles on many of the points above.

Software and code

Policy information about availability of computer code

Data collection	Microsoft Excel Version 16.16.14 was used for patient data collection
Data analysis	Analysis was performed with R version 3.6.1.<br><br>R packages:<br>base packages:<br>grid_3.6.1<br>stats_3.6.1<br>graphics_3.6.1<br>grDevices_3.6.1<br>utils_3.6.1<br>datasets_3.6.1<br>methods_3.6.1<br>base_3.6.1<br><br>additional packages:<br>reshape2_1.4.3<br>survival_3.2-13<br>survminer_0.4.9<br>ggpubr_0.2.5<br>magrittr_2.0.3

ggplot2_3.3.2
swimplot_1.2.0
circlize_0.4.9
RColorBrewer_1.1-2
ComplexHeatmap_2.5.1
openxlsx_4.1.4

Flow Jo version 10.8.1

ImageJ version 1.53

For manuscripts utilizing custom algorithms or software that are central to the research but not yet described in published literature, software must be made available to editors and reviewers. We strongly encourage code deposition in a community repository (e.g. GitHub). See the Nature Portfolio guidelines for submitting code & software for further information.

Data

Policy information about availability of data

All manuscripts must include a data availability statement. This statement should provide the following information, where applicable:

- Accession codes, unique identifiers, or web links for publicly available datasets
- A description of any restrictions on data availability
- For clinical datasets or third party data, please ensure that the statement adheres to our policy

The data that support the findings of this study are not openly available due to patient privacy and are available from the corresponding author upon reasonable request. They are stored on the controlled access repository of the University Hospital Mannheim.

Human research participants

Policy information about studies involving human research participants and Sex and Gender in Research.

Reporting on sex and gender	Both sex and gender were registered based on self reporting and all among our 8 patients there were no differences in reported sex and gender. We analyzed whether immunogenicity of H3 K27M-vac was associated with sex or gender, but did neither expect nor find an association.
Population characteristics	Covariate-relevant population characteristics are stated in Figure 1a, Extended Data Table 1 and 2 :<br>Age at start of vaccination (safety and immunogenicity might depend on patient age)<br>Sex (safety and immunogenicity might depend on patient sex)<br>HLA Type (safety and immunogenicity might depend on patient HLA type)<br>tumor diameter at start of vaccination as judged by product of maximal orthogonal tumor diameter on T1 weighted MRI with contrast enhancement (safety and immunogenicity might depend on tumor diameter)<br>tumor localization (safety and immunogenicity might depend tumor localization)<br>time of initial diagnosis (PFS and OS might depend tumor localization)<br>Karnofsky Performance Index (PFS and OS might depend tumor localization)<br>Oral dexamethasone intake at start of therapy (safety and immunogenicity might depend dexamethasone intake at start of therapy)<br>Extend of resection at initial diagnosis (PFS and OS might depend tumor localization)<br>Dose and fractionation scheme of prior radiation (PFS and OS might depend dose and fractionation scheme of prior radiation)<br>Dose and type of prior chemotherapy (PFS and OS might depend dose and type of prior chemotherapy)<br>co-morbidities (PFS and OS might depend on co-morbidities)
Recruitment	Patients were recruited by referral from clinical neurooncologists. All patients eligible for treatment as determined by the treatment plan were extensively informed about the possibility of treatment with H3K27M-vac and potential therapeutic alternatives. Only patients who provided written informed consent after sufficient time for reflection were treated with H3K27M-vac. We therefore cannot exclude self-selection bias which might be responsible for the favorable overall survival of the entire patient cohort together with the required Karnofski performance score of above 60%. It seems unlikely that self selection had a meaningful influence on safety and immunogenicity.
Ethics oversight	Institutional review board (Ethikkommission) University Hospitals Mannheim, Heidelberg University.

Note that full information on the approval of the study protocol must also be provided in the manuscript.

Field-specific reporting

Please select the one below that is the best fit for your research. If you are not sure, read the appropriate sections before making your selection.

☒ Life sciences ☐ Behavioural & social sciences ☐ Ecological, evolutionary & environmental sciences

For a reference copy of the document with all sections, see nature.com/documents/nr-reporting-summary-flat.pdf

Life sciences study design

All studies must disclose on these points even when the disclosure is negative.

Sample size	We limited the sample size to eight adult patients with recurrent, histologically confirmed H3K27M+ DMG after standard therapy options and not eligible to be enrolled in the currently ongoing multicenter, phase 1 clinical trial (NCT04808245), because therapy was given on a compassionate use basis and not as part of a clinical trial.<br>The compassionate use program was limited to a small number of patients and eight adult patients were judged to be sufficient to preliminarily assess safety and immunogenicity of H3K27Mvac in patients with progressive DMG.
Data exclusions	No data were excluded from the study.
Replication	To ensure reproducibility of our findings we carefully layed out a treatment plan, determined and reported patient characteristics that might be relevant to the effects studied. Furthermore, we described all experimental procedures in detail and will be happy to refine reporting if reviewers have questions or suggestions for improvement. All experiments were performed once unless otherwise reported. PLA was performed independently twice as indicated in legends of Figure 4 and Extended Figure 5. The attempt of replication was successful.
Randomization	Since all patients received H3K27M-vac randomization did not apply. We extensively determined and reported patient characteristics and tested for several potential covariates such as<br>age (p=0.60), sex (p=0.46), KPS (p=0.75), extent of resection (p=0.94), tumor size (p=0.21), time from histological diagnosis to start of vaccination (p=0.06), concomitant anti-PD1 treatment (p=0.47), dexamethasone intake at baseline (p=0.15) or HLA allelotype. Nontheless it should be noted that due to the limited number of patients absence of association does not exclude covariates.
Blinding	This was an open label treatment with both patients and treating physcicians being aware of the treatment with H3K27M-vac. Efficacy assessment was explicitly not part of the analysis and would require a blinded randomized controlled clinical trial. We report the experiences with a limited number of patients threated on a compassionate use basis.

Reporting for specific materials, systems and methods

We require information from authors about some types of materials, experimental systems and methods used in many studies. Here, indicate whether each material, system or method listed is relevant to your study. If you are not sure if a list item applies to your research, read the appropriate section before selecting a response.

Materials & experimental systems

n/a	Involved in the study	
☐	☒	Antibodies
☒	☐	Eukaryotic cell lines
☒	☐	Palaeontology and archaeology
☒	☐	Animals and other organisms
☐	☒	Clinical data
☒	☐	Dual use research of concern

Methods

n/a	Involved in the study	
☒	☐	ChIP-seq
☐	☒	Flow cytometry
☐	☒	MRI-based neuroimaging

Antibodies

Antibodies used	Elispot: anti-human IFN (1-D1K, Mabtech, 3420-3-250), anti-human IFN (7-B6-1, Mabtech, 3420-6-250);<br>Flow Cytometry: CD3-Fitc (HIT3a, BD, 561802), CD4-BV605 (clone SK3, BD, 565998), CD8-PerCP-Cy5.5 (clone RPA-T8, Invitrogen, 45-0088-42), CD45RA-APC-H7 (clone 5H9, BD, 561212), CCR7-BV711 (clone 150503, BD, 566602), PD1-PE (clone EH12.1, BD, 560795), CD25-BV605 (clone 2A3, BD, 562660), HLA-DR-APC-H7 (clone G46-6, BD, 561358), IFNγ-BV421 (clone, 4S.B3, BD, 564791), TNFα-APC (clone, Mab11, Biolegend, 502912) , FoxP3-PE (clone 259/C7, BD, 560046).<br>PLA: mouse monoclonal anti-human GFAP (1:2000, Cell signal, 3670), rabbit polyclonal anti-human IBA-1 (1:100, Wako, 019-19741), donkey anti-mouse Alexa Fluor 488 (1:300, Molecular Probes, Invitrogen, A-21202) and donkey anti-rabbit Alexa Fluor 488 (1:300, Molecular Probes, Invitrogen, A-21206)
Validation	all antibodies are ROA reagents. Flow cytometry antibodies were titrated for optimal signal to noise rations.<br>Validation of FACS Antibodies:<br>CD3-Fitc (HIT3a, BD, 561802):<br>Barclay NA, Brown MH, Birkeland ML, et al, ed. The Leukocyte Antigen FactsBook. San Diego, CA: Academic Press; 1997. (Biology)<br>Beverley PC, Callard RE. Distinctive functional characteristics of human "T" lymphocytes defined by E rosetting or a monoclonal anti-T cell antibody. Immunol. 1981; 11(4):329-334. (Biology)<br>Knapp W, Dorken B, Rieber EP, et al, ed. Leucocyte Typing IV. New York: Oxford University Press; 1989:1-1208. (Biology)<br>Lanier LL, Allison JP, Phillips JH. Correlation of cell surface antigen expression on human thymocytes by multi-color flow cytometric analysis: implications for differentiation. J Immunol. 1986; 137(8):2501-2507. (Biology)<br>McMichael AJ, Beverly PCL, Gilks W, et al, ed. Leukocyte Typing III: White Cell Differentiation Antigens. New York: Oxford University Press; 1987. (Biology) Schlossman SF, Boumsell L, Gilks W, et al, ed. Leukocyte Typing V: White Cell Differentiation Antigens. New York: Oxford University Press; 1995. (Clone-specific)

CD4-BV605 (clone SK3, BD, 565998):
Bernard A, Boumsell L, Hill C. Joint report of the first international workshop on human leucocyte differentiation antigens by the investigators of the participating laboratories. In: Bernard A, Boumsell L, Dausset J, Milstein C, Schlossman SF, ed. Leucocyte Typing. New York, NY: Springer-Verlag; 1984:9-108.
Engleman EG, Benike CJ, Glickman E, Evans RL. Antibodies to membrane structures that distinguish suppressor/cytotoxic and helper T lymphocyte subpopulations block the mixed leukocyte reaction in man. J Exp Med. 1981; 154(1):193-198. (Clone-specific: Functional assay, Inhibition).
Evans RL, Wall DW, Platsoucas CD, et al. Thymus-dependent membrane antigens in man: inhibition of cell-mediated lympholysis by monoclonal antibodies to TH2 antigen. Proc Natl Acad Sci U S A. 1981; 78(1):544-548. (Immunogen: Flow cytometry, Inhibition).
Reichert T, DeBruyere M, Deneys V, et al. Lymphocyte subset reference ranges in adult Caucasians. Clin Immunol Immunopathol. 1991; 60(2):190-208. (Biology).
Sattentau QJ, Dalgleish AG, Weiss RA, Beverley PC. Epitopes of the CD4 antigen and HIV infection. Science. 1986; 234(4780):1120-1123. (Biology).
Wood GS, Warner NL, Warnke RA. Anti–Leu-3/T4 antibodies react with cells of monocyte/macrophage and Langerhans lineage. J Immunol. 1983; 131(1):212-216. (Biology).

CD8-PerCP-Cy5.5 (clone RPA-T8, Invitrogen, 45-0088-42):
Vardam-Kaur T, Pathangey LB, McCormick DJ, Bergsagel PL, Cohen PA, Gendler SJ. Multipeptide stimulated PBMCs generate TEM/TCM for adoptive cell therapy in multiple myeloma. Oncotarget. 2021 Sep 28;12(20):2051-2067.

CD45RA-APC-H7 (clone 5H9, BD, 561212):
Barclay NA, Brown MH, Birkeland ML, et al, ed. The Leukocyte Antigen FactsBook. San Diego, CA: Academic Press; 1997.
Johnson P, Maiti A, Ng DHW. CD45: A family of leukocyte-specific cell surface glycoproteins. In: Herzenberg LA, Weir DM, Herzenberg LA, Blackwell C , ed. Weir's Handbook of Experimental Immunology, Vol 2. Cambridge: Blackwell Science; 1997:62.1-62.16.
Knapp W. W. Knapp .. et al., ed. Leucocyte typing IV : white cell differentiation antigens. Oxford New York: Oxford University Press; 1989:1-1182.
Picker LJ, Treer JR, Ferguson-Darnell B, Collins PA, Buck D, Terstappen LW. Control of lymphocyte recirculation in man. I. Differential regulation of the peripheral lymph node homing receptor L-selectin on T cells during the virgin to memory cell transition. J Immunol. 1993; 150(3):1105-1121. (Biology).
Schwinzer R. Cluster Report: CD45/CD45R. In: Knapp W. W. Knapp .. et al., ed. Leucocyte typing IV : white cell differentiation antigens. Oxford New York: Oxford University Press; 1989:628-634.

CCR7-BV711 (clone 150503, BD, 566602):
Birkenbach M, Josefsen K, Yalamanchili R, Lenoir G, Kieff E. Epstein-Barr virus-induced genes: first lymphocyte-specific G protein-coupled peptide receptors. Nature. 1993; 67(4):2209-2220. (Biology).
Burgstahler R, Kempkes B, Steube K, Lipp M. Expression of the chemokine receptor BLR2/EBI1 is specifically transactivated by Epstein-Barr virus nuclear antigen 2. Biochem Biophys Res Commun. 1995; 215(2):737-743. (Biology). View Reference
Kim CH, Pelus LM, White JR, Broxmeyer HE. Macrophage-inflammatory protein-3 beta/EBI1-ligand chemokine/CK beta-11, a CC chemokine, is a chemoattractant with a specificity for macrophage progenitors among myeloid progenitor cells. J Immunol. 1998; 161(5):2580-2585. (Biology).
Loria MP, Dambra P, Capuzzimati L, et al. Cytokine/Chemokine HLDA8 Workshop panel report: Analysis of receptors on lymphocytes from cord blood, normal and asthmatic subjects, and HIV positive patients. Cell Immunol. 2005; 236(1-2):105-109. (Clone-specific: Flow cytometry).
Sallusto F, Lenig D, Forster R, Lipp M, Lanzavecchia A. Two subsets of memory T lymphocytes with distinct homing potentials and effector functions. Nature. 1999; 401(6754):708-712. (Biology).
Schweickart VL, Raport CJ, Godiska R, et al. Cloning of human and mouse EBI1, a lymphoid-specific G-protein-coupled receptor encoded on human chromosome 17q12-q21.2. Genomics. 1994; 23(3):643-650. (Biology).
Yanagihara S, Komura E, Nagafune J, Watarai H, Yamaguchi Y. EBI1/CCR7 is a new member of dendritic cell chemokine receptor that is up-regulated upon maturation. J Immunol. 1998; 161(6):3096-3102. (Biology).
Yoshida R, Imai T, Hieshima K, et al. Molecular cloning of a novel human CC chemokine EBI1-ligand chemokine that is a specific functional ligand for EBI1, CCR7. J Biol Chem. 1997; 272(21):13803-13809. (Biology).
Yoshida R, Nagira M, Imai T, et al. EBI1-ligand chemokine (ELC) attracts a broad spectrum of lymphocytes: activated T cells strongly up-regulate CCR7 and efficiently migrate toward ELC. Int Immunol. 1998; 10(7):901-910. (Biology).
Yoshida R, Nagira M, Kitaura M, Imagawa N, Imai T, Yoshie O. Secondary lymphoid-tissue chemokine is a functional ligand for the CC chemokine receptor CCR7. J Biol Chem. 1998; 273(12):7118-7122. (Biology).

PD1-PE (clone EH12.1, BD, 560795):
Bennett F, Luxenberg D, Ling V, et al. Program death-1 engagement upon TCR activation has distinct effects on costimulation and cytokine-driven proliferation: attenuation of ICOS, IL-4, and IL-21, but not CD28, IL-7, and IL-15 responses. J Immunol. 2003; 170(2):711-718. (Biology).
Carter L, Fouser LA, Jussif J, et al. PD-1:PD-L inhibitory pathway affects both CD4(+) and CD8(+) T cells and is overcome by IL-2. Eur J Immunol. 2002; 32:634-643. (Biology).
Dorfman DM, Brown JA, Shahsafaei A, Freeman GJ. Programmed death-1 (PD-1) is a marker of germinal center-associated T cells and angioimmunoblastic T-cell lymphoma. Am J Surg Pathol. 2006; 30:802-810. (Clone-specific: Immunohistochemistry). View Reference
Freeman GJ, Long AJ, Iwai Y, et al. Engagement of PD-1 immunoinhibitory receptor by a novel B7 family member leads to negative regulation of lymphocyte activation. J Exp Med. 2000; 192:1027-1034. (Biology).
Latchman Y, Wood CR, Chernova T, et al. PD-L2 is a second ligand for PD-1 and inhibits T cell activation. Nat Immunol. 2001; 2(3):261-268. (Biology).
Nishimura H, Minato N, Nakano T, Honjo T. Immunological studies on PD-1 deficient mice: implication of PD-1 as a negative regulator for B cell responses. Int Immunol. 1998; 10(10):1563-1572. (Biology).

CD25-BV605 (clone 2A3, BD, 562660):
Bach JF. Regulatory T cells under scrutiny. Nat Rev Immunol. 2003; 3(3):189-198. (Biology).
Greene WC, Leonard WJ. The human interleukin-2 receptor. Annu Rev Immunol. 1986; 4:69-95. (Biology).
Jackson AL, Matsumoto H, Janszen M, Maino V, Blidy A, Shye S. Restricted expression of p55 interleukin 2 receptor (CD25) on normal

T cells. Clin Immunol Immunopathol. 1990; 54(1):126-133. (Biology).
Lando Z, Sarin P, Megson M, et al. Association of human T-cell leukaemia/lymphoma virus with the Tac antigen marker for the human T-cell growth factor receptor. Nature. 1983; 305(5936):733-736. (Biology).
Leonard WJ, Depper JM, Uchiyama T, Smith KA, Waldmann TA, Greene WC. A monoclonal antibody that appears to recognize the receptor for human T-cell growth factor; partial characterization of the receptor. Nature. 1982; 300(5889):267-269. (Biology).
Ng WF, Duggan PJ, Ponchel F, et al. Human CD4(+)CD25(+) cells: a naturally occurring population of regulatory T cells. Blood. 2001; 98(9):2736-2744. (Biology).
Rambaldi A, Young DC, Herrmann F, Cannistra SA, Griffin JD. Interferon-gamma induces expression of the interleukin 2 receptor gene in human monocytes. Eur J Immunol. 1987; 17(1):153-156. (Biology).
Robb RJ, Greene WC, Rusk CM. Low and high affinity cellular receptors for interleukin 2. Implications for the level of Tac antigen. J Exp Med. 1984; 160(4):1126-1146. (Biology).
Schwarting R, Stein H. Cluster report: CD25. In: Knapp W. W. Knapp .. et al., ed. Leucocyte typing IV : white cell differentiation antigens. Oxford New York: Oxford University Press; 1989:399-403.
Sereti I, Martinez-Wilson H, Metcalf JA, et al. Long-term effects of intermittent interleukin 2 therapy in patients with HIV infection: characterization of a novel subset of CD4(+)/CD25(+) T cells. Blood. 2002; 100(6):2159-2167. (Biology). View Reference
Siegel JP, Sharon M, Smith PL, Leonard WJ. The IL-2 receptor beta chain (p70): role in mediating signals for LAK, NK, and proliferative activities. Science. 1987; 238(4823):75-78. (Biology).
Teshigawara K, Wang HM, Kato K, Smith KA. Interleukin 2 high-affinity receptor expression requires two distinct binding proteins. J Exp Med. 1987; 165(1):223-238. (Biology).
Urdal DL, March CJ, Gillis S, Larsen A, Dower SK. Purification and chemical characterization of the receptor for interleukin 2 from activated human T lymphocytes and from a human T-cell lymphoma cell line. Proc Natl Acad Sci U S A. 1984; 81(20):6481-6485. (Biology).

HLA-DR-APC-H7 (clone G46-6, BD, 561358):
Barclay NA, Brown MH, Birkeland ML, et al, ed. The Leukocyte Antigen FactsBook. San Diego, CA: Academic Press; 1997.
Dieckmann D, Plottner H, Berchtold S, Berger T, Schuler G. Ex vivo isolation and characterization of CD4(+)CD25(+) T cells with regulatory properties from human blood. J Exp Med. 2001; 193(11):1303-1310. (Clone-specific: Flow cytometry).
Ibisch C, Pradal G, Bach JM, Lieubeau B. Functional canine dendritic cells can be generated in vitro from peripheral blood mononuclear cells and contain a cytoplasmic ultrastructural marker.. J Immunol Methods. 2005; 298(1-2):175-82. (Clone-specific).
Kitani A, Chua K, Nakamura K, Strober W. Activated self-MHC-reactive T cells have the cytokine phenotype of Th3/T regulatory cell 1 T cells. J Immunol. 2000; 165(2):691-702. (Clone-specific: Flow cytometry).
Moran TP, Collier M, McKinnon KP, Davis NL, Johnston RE, Serody JS. A novel viral system for generating antigen-specific T cells. J Immunol. 2008; 175(5):3431-3438. (Clone-specific: Flow cytometry).
Sorg RV, Kogler G, Wernet P. Identification of cord blood dendritic cells as an immature CD11c- population. Blood. 1999; 93(7):2302-2307. (Clone-specific: Flow cytometry).

IFNγ-BV421 (clone, 4S.B3, BD, 564791):
Fonteneau JF, Le Drean E, Le Guiner S, Gervois N, Diez E, Jotereau F. Heterogeneity of biologic responses of melanoma-specific CTL. J Immunol. 1997; 159(6):2831-2839. (Clone-specific: Flow cytometry).
Meager A, Parti S, Barwick S, Spragg J, O'Hagan K. Detection of hybridomas secreting monoclonal antibodies to human gamma interferon using a rapid screening technique and specificity of certain monoclonal antibodies to gamma interferon. J Interferon Res. 1984; 4(4):619-625. (Immunogen: Immunoprecipitation, Radioimmunoassay).
Meager A. Characterization of interferons and immunoassays. In: Clemens MJ, Morris AG, Gearing AJH, ed. Lymphokines and Interferons. A Practical Approach. Oxford: IRL Press Ltd; 1987:105-127.
Prussin C, Metcalfe DD. Detection of intracytoplasmic cytokine using flow cytometry and directly conjugated anti-cytokine antibodies. J Immunol Methods. 1995; 188(1):117-128. (Methodology: Flow cytometry).
Rotteveel FT, Kokkelink I, van Lier RA, et al. Clonal analysis of functionally distinct human CD4+ T cell subsets. J Exp Med. 1988; 168(5):1659-1673. (Clone-specific: ELISA).

TNFα-APC (clone, Mab11, Biolegend, 502912):
Rathjen D, et al. 1991. Mol. Immunol. 28:79.
Ablamunits V, et al. 2010. Eur. J. Immunol. 40:2891.
Enr quez J, et al. 2002. Adv. Perit. Dial. 18:177.
Andersson U, et al. 1999. Detection and quantification of gene expression. New York:Springer-Verlag.
Chen H, et al. 2005. J. Immunol. 175:591.
Iwamoto S, et al. 2007. J. Immunol. 179:1449.
Andersson U, et al. 2000. J. Exp. Med. 192:565.
Moormann AM, et al. 1999. J. Infect. Dis. 180:1987.
Zhao XJ, et al. 2003. J. Immunol. 170:2923.
Rieger R, et al. 2009. Cancer Gene Ther. 1:53-64.
Maksaereekul S, et al. 2009. Vaccine. 28:3754

FoxP3-PE (clone 259/C7, BD, 560046):
Brunkow ME, Jeffery EW, Hjerrild KA, et al. Disruption of a new forkhead/winged-helix protein, scurfin, results in the fatal lymphoproliferative disorder of the scurfy mouse. Nat Genet. 2001; 27(1):68-73. (Biology).
Giovanna Roncador et al. Analysis of Foxp3 protein expression in human CD4+CD25+ regulatory Tcells at a single cell level. Eur J Immunol. 2005; 35(Immunogen).
Wildin RS, Ramsdell F, Peake J, et al. X-linked neonatal diabetes mellitus, enteropathy and endocrinopathy syndrome is the human equivalent of mouse scurfy. Nat Genet. 2001; 27(1):18-20. (Biology).

Elispot:
anti-human IFN (1-D1K, Mabtech, 3420-3-250) and anti-human IFN (7-B6-1, Mabtech, 3420-6-250):
Apostolovic D, Grundström J, Kiewiet MBG, Perusko M, Hamsten C, Starkhammar M, Paulie S, van Hage M. Th2-skewed T cells correlate with B cell response to α-Gal and tick antigens in α-Gal syndrome. J Clin Invest. 2023 Mar 15
Foord E, Arruda LCM, Gaballa A, Klynning C, Uhlin M. Characterization of ascites- and tumor-infiltrating γδ T cells reveals distinct repertoires and a beneficial role in ovarian cancer. Sci Transl Med. 2021 Jan 20;13(577)

PLA:
mouse monoclonal anti-human GFAP (1:2000, Cell signal, 3670):
Eng, L.F. et al. (2000) Neurochem. Res. 25, 1439-51.
Goebel, H.H. et al. (1987) Acta. Histochem. Suppl. 34, 81-93.
Jessen, K.R. et al. (1990) Development 109, 91-103.

rabbit polyclonal anti-human IBA-1 (1:100, Wako, 019-19741:
Imai, Y., Ibata, I., Ito, D., Ohsawa, K., & Kohsaka, S.: Biochem. Biophys. Res. Commun., 224(3), 855(1996).
A Novel Geneiba1 in the Major Histocompatibility Complex Class III Region Encoding an EF Hand Protein Expressed in a Monocytic Lineage
Mori, I., Imai, Y., Kohsaka, S., & Kimura, Y.: Microbiol. Immunol., 44(8), 729(2000).
Upregulated expression of Iba1 molecules in the central nervous system of mice in response to neurovirulent influenza A virus infection
Sasaki, Y., Ohsawa, K., Kanazawa, H., Kohsaka, S., & Imai, Y. Biochem. Biophys. Res. Commun., 286(2), 292(2001).
Iba1 is an actin-cross-linking protein in macrophages/microglia.
Ahn, J.H., et al.: Lab. Anim. Res., 28(3), 165 (2012).
Comparison of alpha-synuclein immunoreactivity in the spinal cord between the adult and aged beagle dog
Ide, T., et al.: J. Vet. Med .Sci., 72(1), 99 (2010).
Histiocytic Sarcoma in the Brain of a Cat
Gaige, S., et al.: Neurotoxicology., 34, 135(2013).
c-Fos immunoreactivity in the pig brain following deoxynivalenol intoxication: Focus on NUCB2/nesfatin-1 expressing neurons
Rodriguez-Callejas, J.D. et al.: Front. Aging Neurosci., 8, 315(2016).
Evidence of Tau Hyperphosphorylation and Dystrophic Microglia in the Common Marmoset
Fantin, A., et al.: Blood, 116(5), 829(2010).
Tissue macrophages act as cellular chaperones for vascular anastomosis downstream of VEGF-mediated endothelial tip cell induction

Clinical data

Policy information about clinical studies
All manuscripts should comply with the ICMJE guidelines for publication of clinical research and a completed CONSORT checklist must be included with all submissions.

Clinical trial registration: Since the patient were treated on a compassionate use basis and not as part of a clinical trial, it was not registered on ClinicalTrials.org.

Study protocol: We provided a treatment protocol that outlines the treatment plan for patients treated with H3K27M on a compassionate use basis.

Data collection: Patients received H3K27M-vac between August 2017 and February 2023 at the University Hospitals of Heidelberg and Mannheim. Both medical centers are tertiary care centers with certified cancer centers by the German Cancer Society.

Outcomes: Safety of H3K27M-vac treatment was assessed during patient visits by a specialized neurooncologist by Common Terminology Criteria for Adverse Events (CTCAE) version 4.0. Immunogenicity was assesd by ELISpot as extensively specified in the methods section of the manuscript. MRI assessment including response assessment was done by neuroradiologists according to iRANO criteria as specified in the methods section of the manuscript.

Flow Cytometry

Plots

Confirm that:

☒ The axis labels state the marker and fluorochrome used (e.g. CD4-FITC).

☒ The axis scales are clearly visible. Include numbers along axes only for bottom left plot of group (a 'group' is an analysis of identical markers).

☒ All plots are contour plots with outliers or pseudocolor plots.

☒ A numerical value for number of cells or percentage (with statistics) is provided.

Methodology

Sample preparation: blood was collected in Li-Heparin tubes and processed within 6 hours of venipuncture by ficoll-density gradient centrifugation. Isolated PBMC were cryopreserved in freezing medium containing 10% DMSO and stored in a liquid nitrogen gas phase tank. Upon thawing, PBMC were rested overnight prior to any functional analysis.

Instrument: BD FACS Lyric

Software: BD FACSuite was used for aquisition, FlowJo V10 was used for analysis.

Cell population abundance: cells were analyzed post in vitro expansion and containd predominantly T cells . Viability was >93 % in all cases. Approximatedly 66% of CD3+ T cells were CD4+. Background in unstimulated cells was below 0.04% of CD4+ and total cytokine secreting cells were > 68%. No cytokine secretion was observed in stimulated CD8+ T cells, but both CD4 and CD8 T cells produced IFN and TNF in response to control stimulation (PMA/ionomycine)

Gating strategy

Hierarchical gating: Exclusions of Debris (FSC-A vs SSC-A)/Exclusion of doublets (FSC-A vs FSC-W), Exclusion of dead-cells (SSC-A vs dead-cell-stain)/Definition of T cells (SSC-A vs CD3-Fitc+)/Definition of T cell subsets (CD8-PerpCP-Cy5.5 vs CD4 BV605)/ Identification of cytokine secreting cells (IFNgamma-BV421 vs TNFalpha-APC), gates were set using unstimulated control

☒ Tick this box to confirm that a figure exemplifying the gating strategy is provided in the Supplementary Information.

Magnetic resonance imaging

Experimental design

Design type	Resting state
Design specifications	single measurement block
Behavioral performance measures	n/a, since no behavioral performance measures were performed

Acquisition

Imaging type(s)	structural
Field strength	3 Tesla
Sequence & imaging parameters	3D fluid-attenuated inversion recovery FLAIR (echo time (TE) = 398 ms, repetition time (TR) = 5000 ms, inversion time (TI) = 1800 ms, field-of-view (FOV) = 240 mm, spatial resolution = 0.5 x 0.5 x 0.9 mm)<br><br>Contrast-enhanced 3D magnetization-prepared rapid acquisition gradient-echo (MPRAGE; TE = 2.49 ms, TR = 1900 ms, TI = 900 ms, FOV = 240 mm, spatial resolution = 0.9 x 0.9 x 0.9 mm)
Area of acquisition	whole brain

Diffusion MRI ☐ Used ☒ Not used

Preprocessing

Preprocessing software	n/a, since images were acquired in clinical routine setting. No preprocessing was performed. Images are merely shown for visualization.
Normalization	n/a, since no normalization was performed.
Normalization template	n/a, since no normalization was performed.
Noise and artifact removal	n/a, since no noise and artifact removal was performed.
Volume censoring	n/a, since no volume censoring was performed.

Statistical modeling & inference

Model type and settings	n/a, since no statistical modeling and inference was performed.
Effect(s) tested	n/a, since no statistical modeling and inference was performed.

Specify type of analysis: ☒ Whole brain ☐ ROI-based ☐ Both

Statistic type for inference (See Eklund et al. 2016)	n/a, , since no statistical modeling and inference was performed.
Correction	n/a, , since no statistical modeling and inference was performed.

Models & analysis

n/a	Involved in the study
☒	☐ Functional and/or effective connectivity
☒	☐ Graph analysis
☒	☐ Multivariate modeling or predictive analysis
